# Improving reliability and absolute quantification of human brain microarray data by filtering and scaling probes using RNA-Seq

**DOI:** 10.1186/1471-2164-15-154

**Published:** 2014-02-24

**Authors:** Jeremy A Miller, Vilas Menon, Jeff Goldy, Ajamete Kaykas, Chang-Kyu Lee, Kimberly A Smith, Elaine H Shen, John W Phillips, Ed S Lein, Mike J Hawrylycz

**Affiliations:** Allen Institute for Brain Science, 551 N 34th Street, Seattle, WA 98103 USA

**Keywords:** Allen Brain Atlas, Microarray, RNA-Seq, High-throughput sequencing, Transcriptome profiling, Reliability, Gene expression, Brain

## Abstract

**Background:**

High-throughput sequencing is gradually replacing microarrays as the preferred method for studying mRNA expression levels, providing nucleotide resolution and accurately measuring absolute expression levels of almost any transcript, known or novel. However, existing microarray data from clinical, pharmaceutical, and academic settings represent valuable and often underappreciated resources, and methods for assessing and improving the quality of these data are lacking.

**Results:**

To quantitatively assess the quality of microarray probes, we directly compare RNA-Seq to Agilent microarrays by processing 231 unique samples from the Allen Human Brain Atlas using RNA-Seq. Both techniques provide highly consistent, highly reproducible gene expression measurements in adult human brain, with RNA-Seq slightly outperforming microarray results overall. We show that RNA-Seq can be used as ground truth to assess the reliability of most microarray probes, remove probes with off-target effects, and scale probe intensities to match the expression levels identified by RNA-Seq. These sequencing scaled microarray intensities (SSMIs) provide more reliable, quantitative estimates of absolute expression levels for many genes when compared with unscaled intensities. Finally, we validate this result in two human cell lines, showing that linear scaling factors can be applied across experiments using the same microarray platform.

**Conclusions:**

Microarrays provide consistent, reproducible gene expression measurements, which are improved using RNA-Seq as ground truth. We expect that our strategy could be used to improve probe quality for many data sets from major existing repositories.

**Electronic supplementary material:**

The online version of this article (doi:10.1186/1471-2164-15-154) contains supplementary material, which is available to authorized users.

## Background

RNA-Seq and related sequencing-based technologies are gradually emerging as the preferred method for genome-wide transcriptional analyses, as they provide several potential advantages over hybridization-based microarray technologies [1–5]. Fragment counts from RNA-Seq more reliably track absolute gene expression levels (as measured by quantitative PCR) than the fluorescence- or intensity-based measures obtained using DNA microarrays [2, 3]. Microarray intensities can also be prone to background noise and hybridization saturation, leading to a lower dynamic range than RNA-Seq [1, 2, 4]. Furthermore, as RNA-Seq does not require *a priori* probe selection, it allows unbiased analysis of the entire transcriptome, including measurements of gene isoforms, noncoding RNAs, novel transcripts [4], and base-level transcriptional changes. But RNA sequencing technologies do not always represent the most appropriate strategy for large scale transcriptomics. In particular, comparison between new and historical data sets is often desired, and direct comparisons across platforms can be problematic [6, 7]. Currently, data from thousands of studies on all of the major microarray platforms are publicly available in databases such as ArrayExpress [8] and Gene Expression Omnibus (GEO) [9]. These data have stimulated important advances in many biological areas over the past two decades, including classification of cancer subtypes [10]; identification of gene expression changes in many diseases; drug discovery [11, 12]; and novel insights into the evolution, development, structure, and dysfunction of the human brain [13–16]. Moreover, microarrays and related technologies are still used in the clinic to measure biomarkers for tumor classification, patient diagnosis, patient prognosis, and predicted response to treatment [17–20]. While there are several options, both commercial (i.e, GeneSpring) and open source (i.e., the "affy" and "limma" libraries in R), for microarray analysis and data quality assessment, to the best of our knowledge none take advantage of the improved absolute gene expression measurements from sequencing technologies.

Here we present the largest comparison between microarray and RNA-Seq to date, using samples from the Allen Human Brain Atlas [14, 21], a publicly available gene expression atlas of the human brain with microarray-based genome-wide transcriptional profiling of specific brain regions spanning all major anatomical structures of the adult brain. RNA aliquots from 231 unique samples across two adult human brains previously analyzed using Agilent microarrays were reprocessed using the Illumina Hiseq RNA-Seq technology, sequenced to a depth of 30 million reads. We find that both methods produce highly reproducible gene expression measurements. RNA-Seq performed slightly better in terms of reproducibility of measurements and detection of differential expression between regions as described previously [2, 3]. However, by treating the RNA-Seq as ground truth, we were able to improve microarray results. First, taking advantage of the high variability of gene expression levels across the adult human brain, we were able to identify the most reliable microarray probe for each gene and remove poorly behaving probes. Moreover, intensities for over 80% of probes could be scaled to provide highly reliable, quantitative estimates of absolute gene expression that should be transferable to any experiment using the same microarray. Finally, we propose an extension to our experimental setup which allows it to be applied to a greater number of probes, and across several microarray platforms. In summary, we find that microarray data can be improved by filtering and scaling probes to RNA-Seq expression values using a relatively small number of samples, and that both methods provide reproducible gene-level expression information that can lead to valuable biological insights.

## Results

### Experimental design

**Figure 1:**
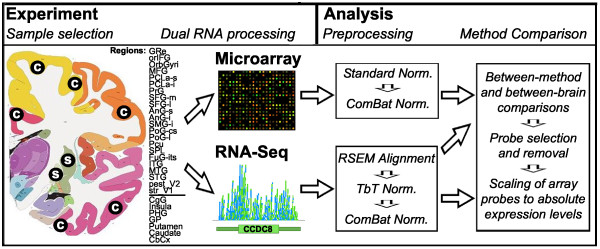
**Experimental design.** RNA from 240 samples spanning 29 neocortical (c) and non-neocortical (s) regions were run using microarray and RNA-Seq in two brains. Gene expression levels were then calculated using comparable strategies. Microarray results were assessed, filtered, and improved using RNA-Seq as ground truth. Details on region selection and preprocessing are available in the Methods and Additional file 3. The source of the microarray image is Guillaume Paumier.

The Allen Human Brain Atlas (http://human.brain-map.org) includes transcriptional profiling data from more than 3500 samples comprising approximately 200 brain regions in six clinically unremarkable adult human brains using custom Agilent DNA microarrays [14, 21]. These arrays include every probe on the Agilent Human GE 44K microarray and approximately 16,000 additional probes. To directly compare the output of transcriptome analysis from microarrays and RNA-Seq across the human brain, we reanalyzed a subset of the same RNA isolates used for microarray analysis using RNA-Seq. A total of 240 samples from 29 matched cortical and subcortical regions in two brains were processed using Illumina HiSeq RNA-Seq technology (Figure 1). In total each brain region was analyzed in eight independent samples, spanning both hemispheres of both brains, with two independent sampling sites per hemisphere (treated as biological replicates). This experiment was designed to facilitate comparisons between biological replicates, between left and right hemispheres, between brains, and across 22 relatively similar neocortical regions and 7 more transcriptionally distinct non-neocortical regions [14]. Overall nine samples were excluded from this analysis—eight technical replicates and one sample that failed quality control—leaving a total of 231 unique samples.

### RNA-Seq data preparation using RSEM alignment followed by TbT and ComBat normalization

Several methods for sequence alignment and gene expression quantification of RNA-Seq data have been developed (for review see [22]) including the Tuxedo Suite [23], RSeqTools [24], and RSEM [25]. These methods each aim to summarize expression levels based on the number of reads that align to each gene, but differ in their treatment of splice junctions and ambiguous sequence alignments. For sequence alignment we used RSEM, which aligns reads to known isoforms and then calculates gene expression as the sum of isoform expression for a given gene, assigning ambiguous reads to multiple isoforms using a maximum likelihood statistical model [25]. The resulting gene expression values are presented as transcripts per million (TPM) after scaling for gene length and for the total number of reads. Our analyses can be reproduced starting from these TPM values using Additional file 1 and data available from the Allen Brain Atlas data portal (http://www.brain-map.org).

Proper normalization of microarray data can remove non-biological differences between samples due to batch effects and differences between arrays. These RNA-Seq data showed minimal batch effects (Additional file 2; supplementary figure legends in Additional file 3), but could potentially still be improved with respect to variability after scaling to the total transcript count. One strategy for doing this is TbT normalization, which scales each sample based on the total number of reads found in genes that are not differentially expressed [26]. This normalization strategy resulted in a slight improvement in data quality when considering cortical vs. non-cortical regions as the two sample groups. Specifically, we see a three-fold decrease in between-sample gene expression variability (based on standard deviation; Additional file 4) and improved between-brain reproducibility, as measured by between-brain correlation of differential expression across brain regions (detailed in next section; increase from R = 0.89 to R = 0.90; Additional file 4). After TbT normalization, we identified a systematic bias between samples from the two brains, in that many genes show a consistent change in expression between brains across many regions assayed. For example, samples from the two brains cluster distinctly for many brain regions (Additional file 2), and furthermore region and brain of origin make up most of the variance between samples (Additional file 4). Whether these between-brain differences are due to real biological differences in brain (i.e., due to age) or technical issues (i.e., due to RNA quality), these systematic differences detract from our ability to compare expression levels between brains, which is one of our primary strategies for assessing biological reproducibility. Therefore, to standardize gene expression data between brains we used ComBat [27], which is an empirical Bayes framework that was designed to remove batch effects from microarray data. In addition to removing the systematic bias between samples from the two brains (Additional file 4), we find that ComBat also improved between-brain reproducibility in our RNA-Seq data (increase from R = 0.90 to R = 0.92; Additional file 4), which justifies our use of this method in this context. Finally, we note that we used a comparable strategy to further normalize the subset of microarray data from the Allen Human Brain Atlas used in this study (Additional file 3), leading to comparable improvements in data quality (Additional file 4).

### RNA-Seq only slightly outperforms microarray based on global reproducibility measures

**Figure 2:**
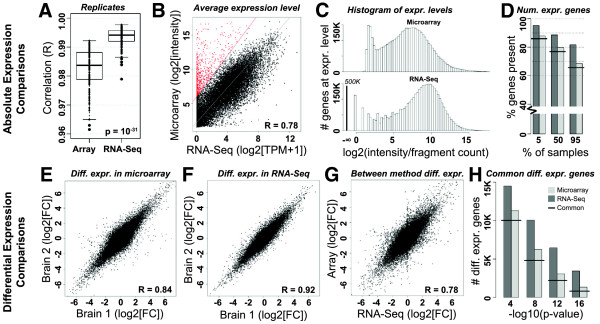
**Microarray and RNA-Seq show highly consistent gene expression metrics. A)** Pearson correlations of absolute expression levels between 115 replicate sample pairs using both methods. **B)** Average log2 expression levels between RNA-Seq (TPM) and microarray (intensity) are strongly correlated. A subset of bright probes (red) show particularly increased intensity in microarray. **C)** Histograms showing distribution of gene expression measures across all samples with microarray (top) and RNA-Seq (bottom). Note the extended leftward tail on the RNA-Seq distribution indicating the lower range sensitivity. **D)** Number of genes called present in microarray (light grey) and RNA-Seq (dark grey) for at least 5%, 50%, and 95% of samples. Horizontal black bars indicate the percentage of overlapping genes called as present using both methods. **E-F)** Correlation of differential expression between brains based on microarray intensity **(E)** and RNA-Seq TPM values **(F)**. Each of 100,000 points shows the log2 fold change of a random gene between two random non-neocortical regions as measured by brain 1 (x-axis) and brain 2 (y-axis). **G)** Correlation of differential expression between methods in the training set (brain 2). Labeling as in **E**, except fold changes correspond to RNA-Seq (x-axis) and microarray (y-axis). **H)** Number of genes differentially expressed between non-neocortical regions based on an ANOVA, for various p-value thresholds. Colors and lines as in **(D)**.

Several strategies for comparing RNA-Seq and microarray technologies have been previously used, including correlation between absolute expression levels, dynamic range assessments, and measurements of differential expression [1–5, 28], but these comparisons typically involved very few samples. In order to quantitatively characterize the quality of gene expression calls from both RNA-Seq and microarray, we performed these and other global reproducibility assessments tailored specifically to our experimental design. We first evaluated the similarity of expression between each pair of biological replicates using Pearson correlation (Figure 2A). RNA-Seq showed a small but significant improvement over microarray (p < 10^-31^; Wilcoxon rank sum test); however, correlations for both methods were quite high (median R = 0.984 vs. R = 0.994), suggesting a high degree of reproducibility when using either method. Next, we directly compared average TPM values for each gene with corresponding average intensities measured by microarray (Figure 2B). These two measures are highly correlated (R = 0.78), at a level consistent with previous studies in liver and kidney ([3]; Spearman = 0.73 and 0.75), nucleus accumbens ([2]; R = 0.698-0.764), and pathogenic bacteria ([28]; Spearman = 0.78 and 0.80). Interestingly, a few hundred genes had at least one (and in some cases all) probes with much higher microarray intensity levels than expected by their TPM values (red dots in Figure 2B), although the converse—genes with high expression in RNA-Seq and low expression with microarray—were very rare. These probes targeted members of several gene families (i.e., histone and keratin genes; Additional file 5) suggesting that they may be more prone to non-specific or off-target hybridization, for example with genes with a high degree of sequence similarity. Furthermore, these probes, which were selected on the basis of their absolute expression differences between methods, also tended not to show consistent differential expression patterns between methods (Additional file 5), suggesting that expression of a subset of probes may not be accurately assessed using microarray (as discussed in detail later).

To compare the dynamic range of both methods, we next plotted a histogram of gene expression levels across all samples in our study (Figure 2C). We note that this result will be highly dependent on sequencing depth, and that our results are based on approximately 30 million reads per sample. RNA-Seq shows higher sensitivity in quantifying genes with very low expression, as shown by the extended leftward tail in the bottom relative to the top plot. Consistent with this finding, more genes were identified as present by RNA-Seq compared to microarray, regardless of the number of samples assessed (Figure 2D). For example, approximately 80% of genes were found by microarray to be expressed in at least half of the samples, compared with approximately 90% by RNA-Seq, with the difference mostly in genes with low expression. However, very few genes identified as absent in RNA-Seq (i.e., no transcript fragments) were called present by microarray, suggesting a relatively low false positive rate in the Agilent present/absent call.

Most genes show expression patterns between brain regions that are highly consistent among individuals [14]. To assess between-brain consistency in these data, we selected 100,000 random genes and 100,000 pairs of randomly selected non-neocortical brain regions in both brains, identified the log2 fold change of each gene between the corresponding pairs of regions, and then plotted these values between brains (Figure 2E-F). Using this strategy, we found highly correlated expression patterns between brains for both microarray (R = 0.84) as well as RNA-Seq (R = 0.92). We next directly compared the magnitude of differential gene expression between methods using the same strategy (Figure 2G). As with absolute expression levels, differential expression fold changes between methods are highly correlated (R = 0.78), at a level consistent with previous studies (Spearman = 0.73 between liver and kidney, for example [3]). These correlations are not as significant as between brains (Figure 2E), despite the fact that RNA from the same RNA samples were used in both methods, supporting results from previous studies that comparisons across platforms can be problematic [6, 7]. Finally, to identify specific genes showing significantly different patterns of expression between non-neocortical brain regions, we performed ANOVA on all samples from these areas. At several p-value thresholds we identified a highly overlapping set of differentially expressed genes, with more genes reaching significance using RNA-Seq than microarray (Figure 2H). Collectively, these global metrics show that, although Illumina sequencing technologies slightly outperform Agilent microarrays by all of these metrics, both methods can consistently and reproducibly evaluate expression levels in the adult human brain for a large percentage of genes.

### Reproducibility dependent on gene expression level and gene size

**Figure 3:**
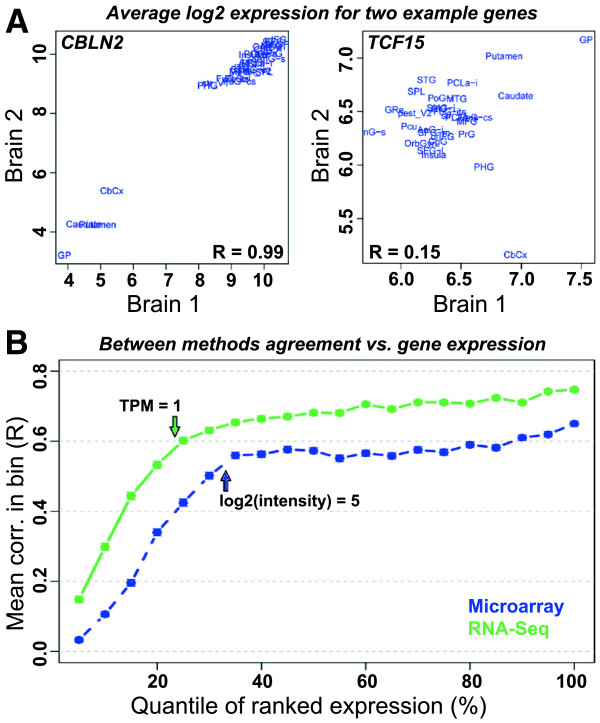
**Gene expression reproducibility is dependent on expression level. A)** Example genes showing good (*CBNL2*, left) and poor (*TCF15*, right) reproducibility using microarray. Reproducibility is defined here based on the between-brain correlation of a gene on average log2(intensity) values in each of the 29 brain regions. **B)** There is a strong relationship between expression level and reproducibility for genes with low expression. Genes were sorted from lowest to highest expression and divided into 20 bins based on expression, which each represent 5% of array genes (x-axis). Each point shows the average between-brain correlation (as in A) for all genes in that bin (y-axis), as measured by microarray (blue) and RNA-Seq (green). Arrows indicate approximate average TPM and intensity values below which RNA-Seq (TPM = 1) and microarray (log2(intensity) = 5) become progressively less reliable. Approximately 25% and 33% of genes have expression levels below these thresholds in RNA-Seq and microarray, respectively. The standard error of the mean (SEM) for each bin is smaller than the dot size.

Genes with very low expression cannot necessarily be reliably evaluated with either arrays or sequencing approaches. In microarray, changes in expression of such genes are often indistinguishable from fluctuations in intensity due to background noise [29]. Likewise, expression measures derived from a small number of sequence fragments are subject to Poisson counting error [3, 30, 31]. Thus, while RNA-Seq yields a broader dynamic range and higher percentage of expressed genes, there is no guarantee that the percentage of genes with reproducibly predicted expression levels will be higher using RNA-Seq (for example, see [4]). To quantify this relationship, we first defined a metric of biological reproducibility—defined as the between-brain correlation of average expression level in each region—for each gene separately (Figure 3A). We then sorted genes based on expression level, divided them into twenty bins of equal size, and identified the mean and standard error of the mean (SEM) for each bin. For both technologies we find that genes expressed at very low levels show progressively decreasing reproducibility with decreasing expression level, whereas for more highly expressed genes (TPM > 1 for RNA-Seq; log2[intensity] > 5 for Agilent microarray), reproducibility is much less dependent on expression level (Figure 3B). Furthermore, regardless of the expression level, RNA-Seq appears to produce more consistent gene expression patterns between brains than microarray. Finally, to test the effect of gene size on reproducibility, we repeated the above assessment, this time sorting genes based on transcript length (Additional file 6). Although the effect is more pronounced with RNA-Seq, we find a nearly linear relationship (R = 0.96) between transcript length and biological reproducibility using both methods. This result appears to be due to a combination of technical variability (the number of sheared fragments per transcript increases with increasing transcript size) and biological variability (larger genes tend to be more differentially expressed across the human brain than smaller genes; Additional file 6). The relationship between gene size and biological variability would be an interesting topic for future study.

### RNA-Seq can filter Agilent microarray probes

**Figure 4:**
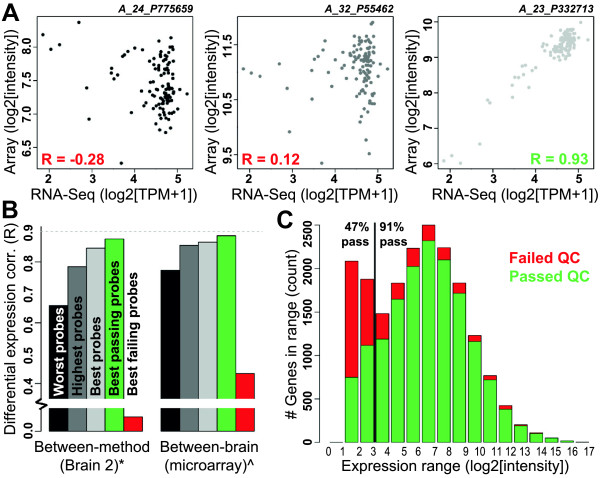
**Probes chosen by RNA-Seq show improved reproducibility metrics. A)** Example gene (ZFR2) with different probes showing the "worst" between-method correlation (left), the "highest" average expression (center) and the "best" between-method correlation (right). Each plot shows the expression level of a microarray probe (y-axis) and the corresponding gene TPM value as measured by RNA-Seq (x-axis). Each dot represents a single sample in our training set (brain 2). Two of these probes would be filtered out as "low quality" using our metric. **B)** Between method (left) and between-brain (right) measures of differential expression correlation when defining microarray genes based on the worst, highest, and best probes (left three bars). Note that correlations in the "highest probes" bars come directly from Figure 2G (*) and Figure 2E (^). The other two bars correspond to the subset of best probes that pass (green) and fail (red) quality control based on our filtering strategy, respectively. Note that the best passing probes have the highest reproducibility. **C)** Genes with low expression are more likely to fail than genes with moderate to high expression. Genes were binned based on expression levels (x-axis) and the number of passing and failing probes is shown for each bin (y-axis). 91% of genes with log2(intensity) > 3 passed, compared to only 47% with lower expression.

Many microarray platforms include multiple probes for a subset of genes. While most such probes show roughly consistent gene expression patterns, when these probes do not agree it is not always obvious which one most accurately reflects gene expression levels. Choosing the probe with the highest expression produces consistent expression levels between experiments (i.e., [32] and this paper); however, such a method only shows that probe expression is reliable and not that the probe uniquely targets the appropriate gene (for example, see Figure 2b and Additional file 5). We hypothesized that choosing optimal probes based on correlation with RNA-Seq TPM calls, which have previously been shown to accurately track absolute gene expression levels [2, 3], should lead to more reproducible microarray results than any strategy based solely on array intensities. To test this we chose the probes with the highest ("best") and lowest ("worst") between-method correlation across samples for each of the 91% of genes with at least two representative probes on the array, and assessed how each set of probes compared with the array-derived, highest expressed probes. As an extreme example, three probes for ZFR2 showed markedly different expression patterns as compared with RNA-Seq (Figure 4A), and in this case correct choice of probe is important. Overall, we find improved between-method reproducibility for our best probes (Figure 4B, left bars), which is expected since our probes were chosen this way. More interestingly, we also see a slight improvement in biological reproducibility between brains (Figure 4B, right bars; R = 0.86 compared with R = 0.85), suggesting that RNA-Seq could be used as a tool for probe selection or at least *a posteriori* analysis. We note that, although only 60% of the best probes were also the most highly expressed, choosing the most highly expressed probe leads to highly biologically reproducible results, as previously shown [32].

In addition to choosing the best probe for each gene, this strategy can be used to assign each probe with a quality score (or pass/fail call) based on reproducibility, which could, for example, help eliminate genes from the analysis in which all probes show potential off-target effects or non-specific binding. In this case we score each probe based on the correlation, defining all probes with significant positive correlation as passing (Figure 4A). After correcting for multiple comparisons (q < 0.1), 82% of genes have at least one passing probe on the array, a number that decreases only to 68% if we consider as few as 16 carefully selected samples in the analysis (Additional file 7). After omitting the set of best probes that failed quality control, the remaining genes show marked improvements in between-method and between-brain reproducibility (Figure 4B; green bars; R = 0.87 vs. 0.85 and R = 0.88 vs. 0.86, respectively). Given our previous result showing that genes with low expression tended to show poor biological reproducibility (Figure 3B), we next compared the expression levels of our best probes that passed compared with those that failed (Figure 4C). Around half the probes with log2(intensity) < 3 passed, compared with more than 90% of probes with log2 (intensity) > 3, suggesting that some probes likely fail because the probe itself is bad, whereas other probes may be properly targeting their corresponding gene, but that gene is not expressed in the brain and therefore the between-method reproducibility cannot be accurately assessed. Strategies for recovering this second class of failed probes will be discussed later.

### RNA-Seq can be used to improve microarray quality by scaling probes

**Figure 5:**
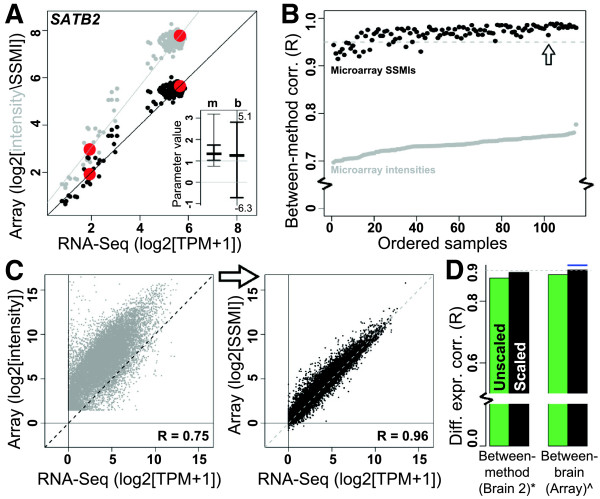
**Scaling of microarray probes by RNA-Seq leads to improved biological reproducibility. A)** Strategy to convert intensity levels of all probes to sequencing scaled microarray intensities (SSMIs) using samples from brain 2. SATB2 is shown as an example. 5^th^ and 95^th^ quantiles (red dots) are compared between methods, and microarray intensities are scaled linearly such that these quantiles align. Grey and black dots show expression of a sample in brain 2 for both methods before and after scaling, respectively. Inset shows the range of slope (m) and intercept (b) parameters across all probes (25%, 50%, and 75% quantiles shown in bold; 5% and 95% quantiles shown in light lines or enumerated if off the plot). **(B** **-C)** After scaling (black dots), all samples in brain 1 show markedly improved between-method correlation of absolute expression levels compared with before (grey dots). This result holds for all 115 samples in brain 1 **(B)**. A single example is shown in **C** (corresponding to the arrow in B; labeling as in Figure 2B). Diagonal dotted line indicates perfect agreement of absolute expression levels (y = x). **D)** SSMIs show improved reproducibility between methods based on between-method (left; * = compare with Figure 2G) and between-brain (right; ^ = compare with Figure 2E) differential expression measures (compare with Figure 4C). The blue line indicates the between-brain correlation as measured by RNA-Seq (Figure 2F), which is now only slightly higher (ΔR=0.02) than in microarray.

Microarray probes tend to measure relative gene expression levels more accurately than absolute levels. However, at non-extreme intensities (where the effects of background noise and oversaturation can mostly be ignored), the relationship between probe intensities and gene expression levels identified through other experimental strategies is nearly linear [33]. We therefore hypothesized that, by using a simple linear transformation, it should be possible to scale probe intensities so that they more accurately reflect absolute expression levels. To calculate such values—which we refer to as "sequencing scaled microarray intensities" or "SSMIs"—we tried several approaches (see Additional file 3). For these data the most effective was a quantile-based approach, where we identified the 5^th^ and 95^th^ quantile of expression for each microarray probe (using intensity) and for the corresponding RNA-Seq gene (using TPM), and then linearly scaled the microarray intensities so that these values align with TPMs (Figure 5A). We performed this scaling strategy using only samples from brain 2, and reserved brain 1 as an independent test set. SSMIs were only calculated for probes passing our quality control assessment, as discussed above. Most probes showed a relatively small range of slopes (m; 50% between 1 and 2) and required a small negative offset (b > 0), suggesting that microarray intensity can be used as a rough approximation for absolute expression levels after adjusting for background, but that the relationship is not identical from probe to probe (Figure 5A, inset). Scaling parameters and probe quality control measurements are provided in Additional file 8.

To test whether SSMIs provide more biologically reproducible results than corresponding intensity scores, we repeated all of our quantitative assessments (see Figures 2 and 3) using SSMIs for the set of optimal probes chosen by RNA-Seq (see Figure 4). As hypothesized, absolute expression levels show a dramatic improvement in reproducibility between RNA-Seq and microarray, with Pearson correlations increasing in many cases to R > 0.95 (Figure 5B). For example, while many microarray probe intensities overestimate gene expression levels by several orders of magnitude, SSMIs for nearly all probes much more closely match TPM values determined by RNA-Seq (Figure 5C). It is important to emphasize that we see these improvements in the test set (brain 1) using scaling parameters calculated using an independent training set (brain 2). Along the same lines, between-method measures of consistency based on differential expression show similar improvements, in both the training set (brain 2; Figure 5D) and test set (brain 1). Furthermore, differential expression fold change correlations between brains based on SSMIs (R = 0.90) are nearly as high as those based on RNA-Seq TPMs (R = 0.92; Figure 5D), suggesting that after probe selection, filtering, and scaling, microarrays can nearly match sequencing technologies in certain measures of biological reproducibility. Comparable improvements can be found if we generate scaling parameters with as few as 16 samples (Additional file 7).

**Figure 6:**
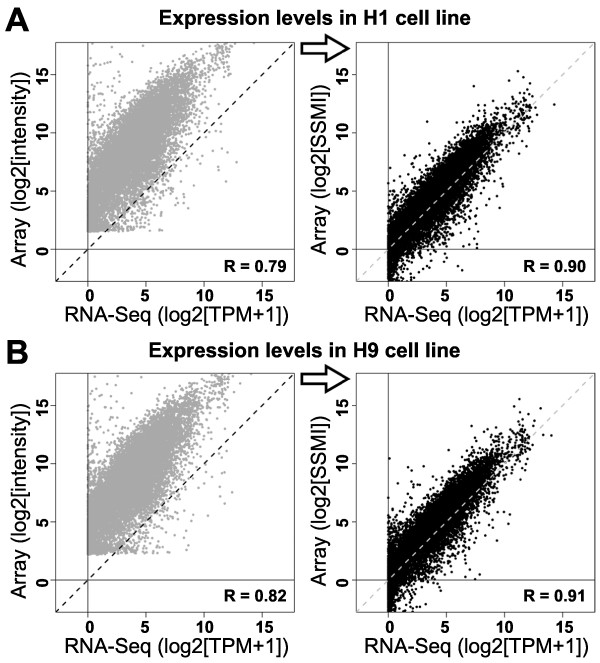
**Scaling parameters generated in human brain also improve measurements of absolute expression levels in human hESC lines.** Improved between-method correlation of absolute expression levels is found in H1 **(A)** and H9 **(B)** human hESC lines after scaling using parameters identified in brain. Each point shows expression levels for a single gene in microarray (y-axis) compared with RNA-Seq (x-axis). Labeling as in Figure 5C.

Finally, to test whether our quantile-based scaling is applicable to gene expression intensities derived from other tissue, we processed RNA from two pluripotent human embryonic stem cell (hESC) lines (H1 and H9; Additional file 3) using both microarray and RNA-Seq, which were used to assess, but not to improve, microarray quality. Following the same computational strategy and using the same scaling parameters derived from brain 2 above, we scaled microarray intensities from these cell lines, and compared both measures of microarray gene expression to TPM values based on RNA-Seq. As with brain, SSMIs in both undifferentiated hESC lines show significantly improved correlation with RNA-Seq relative to unscaled intensities that much more closely map to RNA-Seq derived absolute intensities (Figure 6). Comparable results were found after differentiating these hESC lines for up to 54 days to generate cortical neurons (data not shown; Additional file 3). Although the Allen Human Brain Atlas and the hESC lines were processed at the same site, we note that different methodologies were used for tissue processing, and that both the microarrays and RNA-Seq for these data sets were processed off-site at different locations (Methods). These results suggest that scaling parameters derived from a single experiment can be applied to other experiments utilizing the same array platform to improve array quality. More generally, we find that RNA-Seq can be used as a tool to evaluate microarray probe quality, filter out bad probes, and improve the utility of microarrays as tools to measure absolute gene expression levels. Such filtering appears largely to be experiment independent, suggesting it could be retroactively applied to improve data from thousands of currently available data sets.

### Identifying differentially expressed genes in neocortex using microarray and RNA-Seq

We have previously shown that differences in transcriptional patterns of distinct neocortical areas depend on the distance between these areas, although comparatively few genes show very high levels of differential expression in the neocortex [14]. To assess the extent to which these more subtle expression relationships can be found using RNA-Seq as compared with microarray, we performed ANOVA on all samples from these 22 neocortical areas. RNA-Seq identified 3458 genes differentially expressed (p < 0.05, Bonferroni corrected), compared with 2144 identified using microarray intensities, of which 1121 agree between methods (p ~ 0). An additional 194 genes were identified using SSMIs instead of intensities, including 120 found by RNA-Seq that were not initially identified by microarray, showing that RNA-Seq scaling improved the sensitivity of microarrays to detect differential expression by approximately 10%, even when comparing relatively similar tissue. Thus, despite the relatively mild transcriptional differences between neocortical areas, we find common differentially expressed genes between methods. Collectively, these results demonstrate the biological reproducibility and applicability of both of these genome-wide transcriptional methodologies.

## Discussion

### Optimizing gene expression measurements from extant microarray data

We presented an extensive comparison between Agilent microarray and RNA-Seq using 231 samples from the Allen Human Brain Atlas [14]. Our analysis aimed to assess and potentially improve the quality of extant microarray data, and had three primary goals. First, we showed that Agilent microarrays generate highly reproducible expression measurements for many genes, both when comparing gene expression measurements with RNA-Seq as well as between individual brains. Our results are in line with previous studies comparing RNA-Seq and several different microarray platforms [1–5], in that we identified high correlations of absolute (R = 0.78) and differential (R = 0.78) expression levels between methods. We also demonstrated a high degree of biological reproducibility for genes with at least moderate expression (TPM > 1, log2[intensity] > 5), which progressively decreases for lower expression levels using both methods. Second, we have shown that RNA-Seq can be used to assess microarray probe quality, and that this can be done using a relatively small number of experimental samples. We saw progressively increasing biological reproducibility of gene expression measurements when we initially used these quality scores to identify the best microarray probe for each gene, and subsequently excluded genes from the analysis with no reliable probes.

Third, and most importantly, our study proposes for the first time the use of high throughput sequencing to scale microarray probe intensities to more closely reflect absolute gene expression levels. Previous studies have presented strategies for measuring absolute expression using microarray, for example, by co-hybridizing biological samples with calibrated reference samples on spotted-glass microarrays [34], and have sought to improve array quality by filtering out bad probes from Affymetrix probe sets [35]. The advantage of our strategy is that these scaling parameters appear to be broadly applicable, as those derived from samples run in adult human brain improved the reliability of expression levels identified using the same array in two different hESC lines. In principal, these parameters (Additional file 8) could now be applied to any sample run using our custom array (or the widely-used Agilent 44K Whole Human Genome Microarray) to improve absolute expression quantification for around 80% of genes without needing to perform any additional sequencing. While these scaling parameters may not be optimal for improving data derived from other experimental conditions, the key point is that they can improve the data without the need for performing additional sequencing.

### Microarrays and RNA-Seq both currently used in research and clinical settings

While RNA-Seq will likely replace microarrays in research and clinical settings in the near future due to the improved dynamic range and potential for finding novel transcripts and sequence level variations, microarray data sets are still highly valuable, and application of this method improves their interpretability. Currently, microarray data from thousands of experiments are available in public databases such as GEO [9] and ArrayExpress [8], providing valuable resources for cross-study comparisons between experiments utilizing the same transcriptional method. For example, both the Allen Human Brain Atlas [14, 21] and a companion BrainSpan atlas targeting prenatal human brain (http://www.brainspan.org) utilize the same Agilent array to facilitate between age comparisons, and have provided insight into the structure and function of the human brain. Furthermore, many research and clinical laboratories already have standard operating procedures in place for analyzing microarray data—including the required machinery, storage space, analysis tools, and expertise—which could be augmented in a relatively straightforward manner using our method, and it will take time to transition to sequencing-based strategies. Finally, until quite recently RNA-Seq techniques required more total RNA (100 ng-1 μg) than microarray [4, 36]. In our study this requirement limited the samples that could be included in the experimental design. However, newer RNA-Seq strategies that allow transcriptional profiling from single cells [37, 38] or even single nuclei [39] hold great promise in categorizing and understanding cortical cell types, and at potentially a fraction of the cost of microarrays. Thus in the near term, microarrays and RNA-Seq will both likely be used for high throughout gene expression analysis, and therefore any strategies for improving the accuracy of detecting and corroborating gene expression signal from microarrays will be helpful.

### Limitations and suggested methodological improvements

One limitation of this analysis is that, in order to accurately assess probe quality and define scaling parameters, the variability across samples must be accurately measured. For example, we found that probes targeting genes with very low expression in brain were much more likely to be failed, compared with high expressers, and that such genes also showed less consistent expression patterns between brains. It is likely that with a more diverse tissue panel some of these low-expressing probes would be assessed as higher quality. Another possible limitation is that our scaling parameters derived from brain may not be applicable to other tissues for genes showing a high degree of differential isoform expression. Again, by using a more diverse tissue panel to calculate scaling parameters, we would expect that probes for such genes would not show consistent expression between methods, and would therefore be failed at the quality control step.

Using our current study as a starting point, we propose a methodology that would address these limitations, and could further be used to improve microarray data quality for many publicly available data sets and clinical applications. First, an RNA atlas of gene expression from several highly distinct tissues, organs, and cells lines would be collected, for example by contacting an accredited tissue bank, or in partnership with a related governmental program such as the Genotype-Tissue Expression (GTEx) Project (http://commonfund.nih.gov/GTEx/index). Our results suggest that approximately 16 carefully chosen samples would be sufficient, although increasing the number of samples would moderately improve the power to detect passing probes (Additional file 7). Previously published gene expression atlases on 46 [40] and later 79 [41] such tissues found that nearly 90% of expressed genes also showed some degree of differential expression, and these atlases could be used as filters for determining the most transcriptionally distinct tissues. Second, RNA from all of these tissues would be processed using RNA-Seq and several of the most commonly used microarray platforms in parallel. Considering only the most widely published array platforms for Affymetrix (HG-U133_Plus_2), Illumina (HumanHT-12 V3.0), and Agilent (014850 Whole Human Genome Microarray 4x44K G4112F), data for around 100,000 microarrays are currently hosted by Gene Expression Omnibus [9]. Additional RNA aliquots could be stored for later processing using other microarray platforms. Third, quality assessments and scaling parameters for each probe (or in the case of Affymetrix, each probe set) of each microarray platform would be assessed as described above. Finally, the resulting values could be applied, in principle, to any data utilizing any of the microarray platforms included in this experiment. We expect that our strategy could also facilitate direct comparisons of data collected using different array platforms, although this hypothesis would need to be tested.

## Conclusions

We showed that both Agilent microarrays and RNA-Seq can provide highly reproducible measurements of gene expression in the human brain. Furthermore, for a majority of genes, we were able to quantifiably assess the reproducibility of microarray probes, remove probes with off-target effects, and scale probe intensities to provide highly reliable, quantitative estimates of absolute gene expression levels. The scaling parameters generated using brain tissue appear to be applicable to other tissues, and are provided as a resource to the community. Overall, we calculated SSMIs values for 80% of the approximately 19,000 genes included in our between-method comparison with moderate confidence, and expect that many of the remaining genes could be scaled using a more diverse set of tissues, as proposed.

## Methods

### Post-mortem tissue acquisition and sample processing

Methods for post-mortem tissue acquisition and sample processing have previously been described ([14] and http://help.brain-map.org/display/humanbrain/Documentation). In short, tissue for the Allen Human Brain Atlas was provided by the NICHD Brain and Tissue Bank (Baltimore, MD) and the University of California Irvine Department of Psychiatry and Human Behavior Brain Donor Program (Irvine, CA), under approvals by Institutional Review Boards of the Maryland Department of Health and Hygiene and University of Maryland Baltimore, or the University of California Irvine, respectively, and with consent from next-of-kin. Specimens for microarray and RNA-Seq profiling were 24-year-old (Brain 1) and 39-year-old (Brain 2) African American males. Total RNA from 120 macrodissected samples initially processed for microarray from each brain (as described in [14]) were also processed for RNA-Seq. These samples included biological replicates from left and right hemisphere in 29 brain regions, as well as four technical replicates per brain. Aliquots of the same total RNA isolates generated from macro dissections for microarray were used for sequencing. RNA was sent to Expression Analysis Inc. (EA; Durham, NC) for library preparation and sequencing, of which 250 ng total RNA was input for each run. EA used the Illumina TruSeq library preparation protocol and performed paired-end, 50 bp sequencing on an Illumina HiSeq2000 instrument. The sequencing was run as 6-plex with target of 30 million reads per sample: 25-35 million reads per sample were generated. Processed data is available at the Allen Brain Atlas data portal (http://www.brain-map.org) in the form of gene counts, as well as TPM values.

### RNA-Seq alignment and data normalization

Sequences were aligned to the genome using RNA-Seq by Expectation-Maximization (RSEM) [25] (see Additional file 3). Transcripts (isoforms) were defined using the knownGene table from UCSC Genome Browser ([42]; http://genome.ucsc.edu; hg19, Feb. 2009). Summary expression levels for each gene were calculated in terms of both counts and TPM using this pipeline. Mapped read files were also converted to BAM file format for visualization using GenomeBrowse (Golden Helix, Bozeman, MT).

Microarray data normalization was performed as described on the Allen Human Brain Atlas data portal (http://help.brain-map.org/display/humanbrain/Documentation). In short, data is preprocessed for systematic biases, and quantile normalized to the 75th percentile in each batch. Across batches within each brain, data is normalized by aligning two sets of control samples included in all batches and capturing method related bias is adjusted by a modified quantile normalization method. We note that approximately 2,200 probes failed quality assessments during generation of the Allen Human Brain Atlas and were also excluded from this analysis. Across multiple brains non-biological difference is adjusted again by aligning two sets of control samples. RNA-Seq data was TbT normalized [26] in linear space, as described in the Results, with the differential expression vector defined as TRUE if a sample was from neocortex and FALSE otherwise. Samples were then scaled such that the total log2(TPM) remained unchanged after normalization. Data from both microarray and RNA-Seq were then ComBat normalized [27] in log2 space using a parametric prior, with the batches corresponding to brain of origin. Unless otherwise noted, ComBat normalized data were used for all comparisons.

### Comparisons between microarray and RNA-Seq

The open source R software (http://www.r-project.org/) was extensively used for all analyses and visualizations (Additional file 3). Hierarchical clustering, bar plots showing expression levels, and MDS in two dimensions were strategies used for assessing data quality and evaluating the effect of normalization (Additional file 3). For all between-method comparisons, unless otherwise noted, a single microarray probe with the highest average expression level across regions [32] was selected to represent each of the approximately 19,000 commonly identified genes. A gene was defined as present in microarray if called present by the Agilent software, and in RNA-Seq if at least one fragment was aligned to that gene.

Pearson correlations (R) were calculated in several contexts as measures of consistency or reproducibility throughout (Additional file 3). Differential expression was measured in two ways. As a global measure of biological or technical reproducibility, fold changes of 100,000 random genes between randomly selected region pairs was compared between brains or methods, respectively, using Pearson correlation. Alternatively, a p-value for each gene was calculated by running ANOVA across either the 7 non-neocortical or 22 neocortical regions, representing transcriptionally diverse and relatively similar tissues, respectively. To assess biological reproducibility of gene subsets, genes were sorted based on average expression level or gene size, binned into 20 groups, and then the average and standard deviation of the across-region Pearson correlations for all genes in each bin were calculated.

### Generation of microarray scaling parameters

SSMIs were calculated by performing a linear scaling on microarray intensities from brain 2 in log2 space. Normalized data prior to ComBat normalization were used for this analysis to preserve samples from brain 1 as independent and since ComBat normalized RNA-Seq data could less closely align to absolute expression levels than do TbT normalized data. For each gene, scaling parameters were found by identifying the 5^th^ and 95^th^ percent quantiles of expression in RNA-Seq and microarray and then linearly shifting microarray expression to match these quantiles with RNA-Seq (see Figure 5A). All probes were scaled, but only the probe for each gene with the highest between-method correlation was included in the final comparison between methods. Scaling confidence was estimated using the significance of correlation (a p-value output by the cor.test R function) and converting to q-values as a measure of false discovery rate using the R function qvalue [43]. Probes with q < 0.1 were scaled, and remaining probes were omitted from the analysis. Finally, scaled array intensities were ComBat normalized as described above for comparison with other results.

### Comparison with human hESC lines

RNA from two human pluripotent ESC lines, H1 and H9, or their cortical neuronal progeny derived using directed differentiation, were run on microarray and RNA-Seq (Additional file 3). In short, RNA was generated by lysing cells in RNAeasy buffer (Qiagen) and then following the standard RNAeasy protocol. RNA was then sent to Covance (for microarray) and Expression Analysis (for RNA-Seq). This is in contrast to RNA from brain, which was processed by Cogenics (for microarray) and Covance (for RNA-Seq), as described above. Correlations between methods were calculated both before and after SSMI scaling to demonstrate the effectiveness of these scaling parameters on absolute expression levels in other tissues.

### Availability of supporting data

All data presented in this manuscript are available either at the Allen Institute data portal (http://www.brain-map.org) or as part of Additional file 1. Specifically, microarray data (both raw and normalized intensities), as well as sequence data summarized to the gene level (both fragment counts and TPM values) can be downloaded from the Allen Institute data portal by clicking on the "Human Brain" link and then the "Download" link. Additional file 1 contains the microarray and sequencing data for hESC lines as well as annotated code for Figures 2, 3, 4, 5 and 6.

## Additional files

Description of additional data files

The following additional data are available with the online version of this paper. Additional file 1 is a zip file including code and supporting data required to reproduce our analysis in R. Additional file 2 is a supplementary figure showing that RNA-Seq has minimal batch effects. Additional file 3 is a text document that includes Supplementary Methods and Supplementary Figure Legends. Additional file 4 is a supplementary figure showing that normalization improves the quality of RNA-Seq and microarray data. Additional file 5 is a supplementary figure showing that certain highly-expressed microarray probes do not accurately measure gene expression. Additional file 6 is a supplementary figure showing that gene expression reproducibility is dependent on transcript length. Additional file 7 is a supplementary figure showing that quality control and scaling of microarray probes can be done well with as few as 16 matched RNA-Seq samples. Additional file 8 is a supplementary table listing scaling parameters for the Agilent Microarray.

## Electronic supplementary material

Additional file 1: **Code and data to reproduce analysis.** This zip file contains two code documents and several supporting data files that are required, along with data from the Allen Brain Atlas data portal (http://www.brain-map.org) to reproduce nearly all figures and statistics presented in this manuscript. (ZIP 11 MB)

Additional file 2: **Clustering of RNA-Seq samples after TbT normalization shows minimal batch effects**. A dendrogram that shows samples hierarchically clustered based on the RNA-Seq data. Also shown are bar plots with biological and technical variables. Samples cluster based on brain region and brain of origin, but not batch or other technical variables. (PDF 3 MB)

Additional file 3: **Supplementary methods and supplementary figure legends.** This file contains the supplementary methods for the manuscript followed by supplementary figure legends corresponding to Additional files 2, 4, 5, 6 and 7. (DOCX 28 KB)

Additional file 4: **Normalization improves the quality of RNA-Seq and microarray data.** Several plots showing that RNA-Seq data becomes progressively more consistent and reproducible after TbT normalization, which scales for the total reads, and ComBat normalization, which removes the systematic bias between brains. Microarray data likewise improved. (PDF 18 MB)

Additional file 5: **Many probes with specifically high microarray intensity do not accurately measure gene expression**. Probes with specifically high expression in microarray show poor between-method agreement, suggesting they do not appropriately measure expression of their assigned gene. These probes are also more likely than chance to be part of gene families. (PDF 3 MB)

Additional file 6: **Gene expression reproducibility is dependent on transcript length.** There is a strong linear relationship between gene length—defined based both on the average transcript length for all RefSeq isoforms of the gene as well as for the number of base pairs spanned in the genome—and reproducibility for genes. This is in part because large genes tend to show higher variability in the brain compared with smaller genes. (PDF 6 MB)

Additional file 7: **Microarray quality control and scaling can be accurately done with 16 samples.** The percent of passing probes rapidly improves when using a small number of matched samples, and starts leveling out at around 16 samples. Excellent between-method correlation and improvements in scaling are also seen with 16 samples or fewer. (PDF 3 MB)

Additional file 8: **Parameters and quality metrics for microarray probe scaling.** This table contains the parameters that can be applied to any Agilent Human GE 44K microarray to scale probes so that they more accurately reflect absolute gene expression levels. It also includes quality metrics which can be used as filters, for example, to omit certain probes from consideration. (XLSX 9 MB)

## Abbreviations

TPM: Transcripts per million
SEM: Standard error of the mean
hESC: Human embryonic stem cell
MDS: Multidimensional scaling
SSMI: Sequencing scaled microarray intensity
GTEx: Genotype-Tissue Expression
EA: Expression Analysis
RSEM: RNA-Seq by expectation-maximization
PC: Principal component.
