# Supplementary figures and images for: Improving reliability and absolute quantification of human brain microarray data by filtering and scaling probes using RNA-Seq
Source: BMC Genomics. 2014 Feb 24;15(1):154. doi: 10.1186/1471-2164-15-154 (PMC4007560; doi:10.1186/1471-2164-15-154)

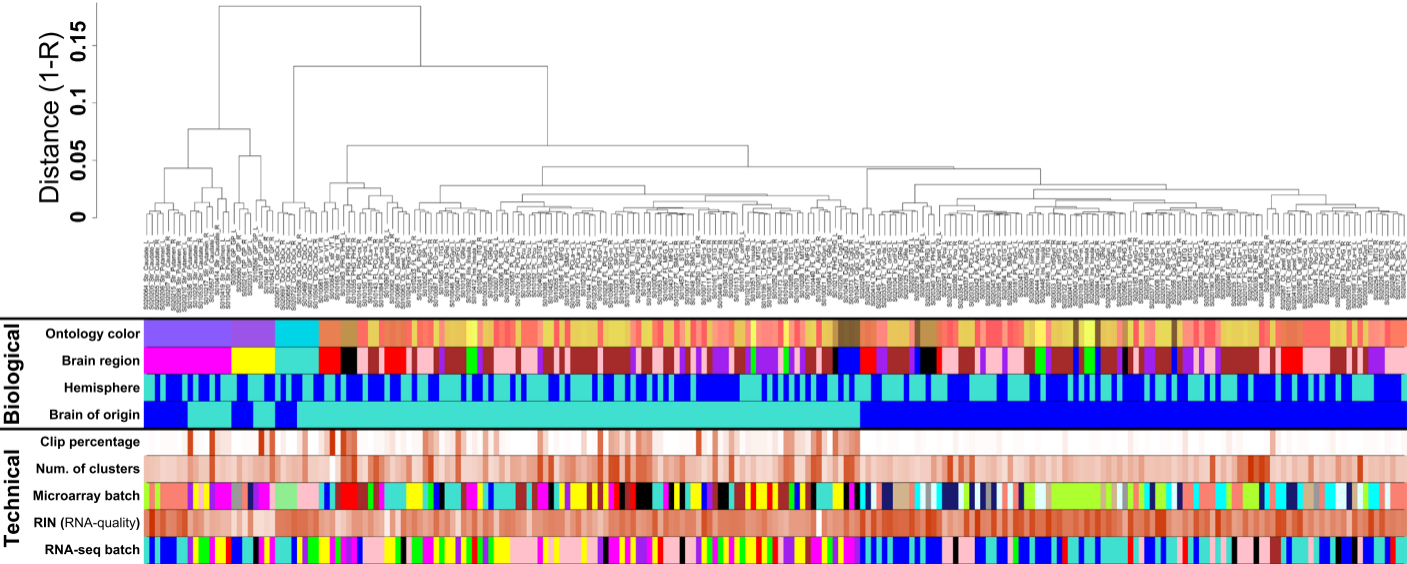

Supplement: Supplementary file 2 — Additional file 2: Clustering of RNA-Seq samples after TbT normalization shows minimal batch effects. A dendrogram that shows samples hierarchically clustered based on the RNA-Seq data. Also shown are bar plots with biological and technical variables. Samples cluster based on brain region and brain of origin, but not batch or other technical variables. (PDF 3 MB) [file 12864_2013_7016_MOESM2_ESM.pdf]

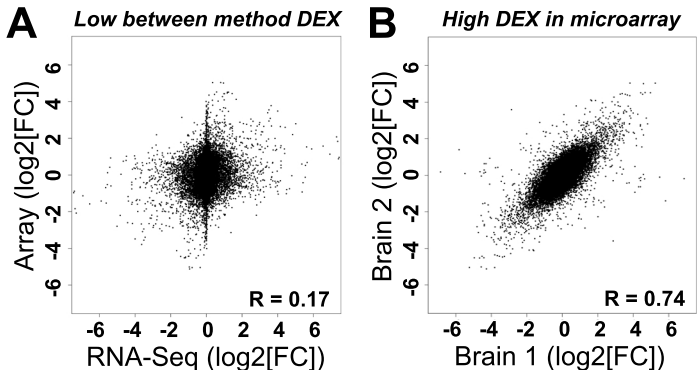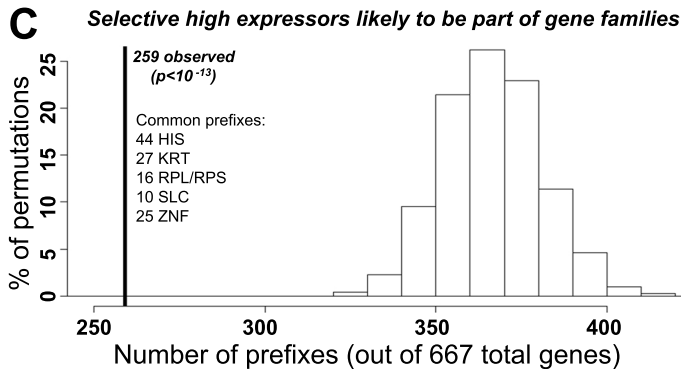

Supplement: Supplementary file 5 — Additional file 5: Many probes with specifically high microarray intensity do not accurately measure gene expression. Probes with specifically high expression in microarray show poor between-method agreement, suggesting they do not appropriately measure expression of their assigned gene. These probes are also more likely than chance to be part of gene families. (PDF 3 MB) [file 12864_2013_7016_MOESM5_ESM.pdf]

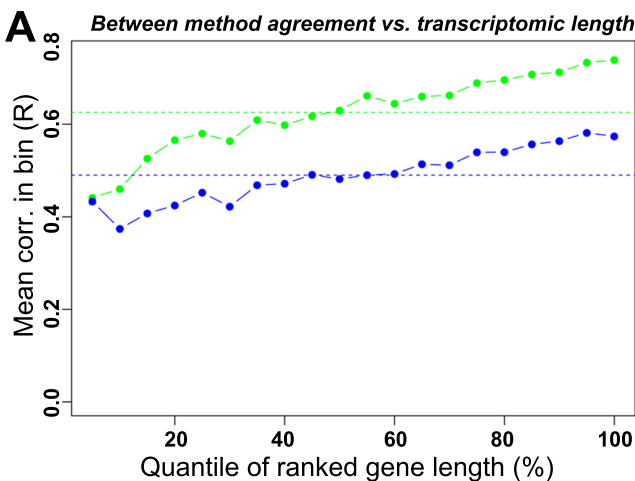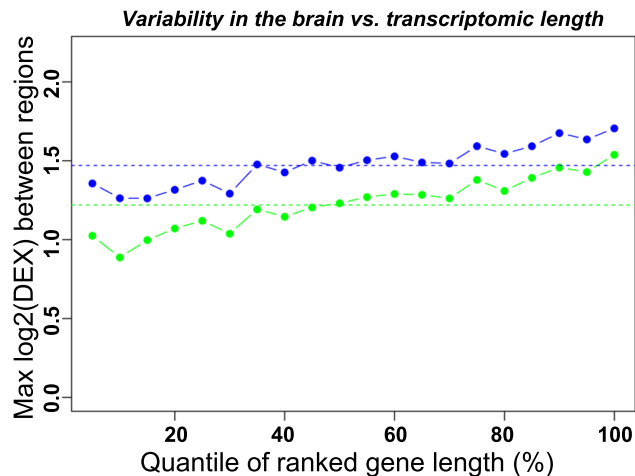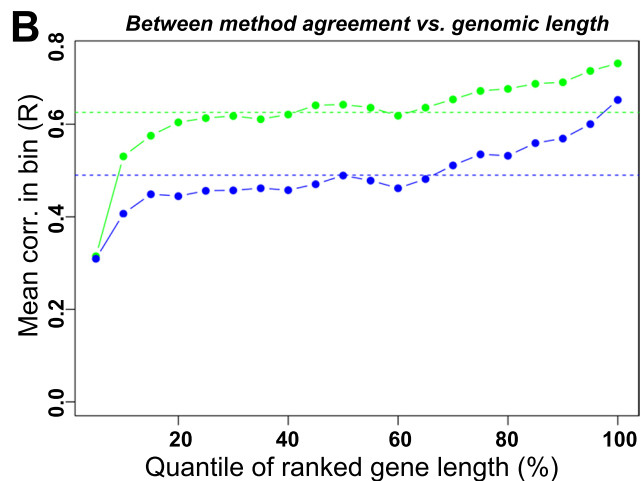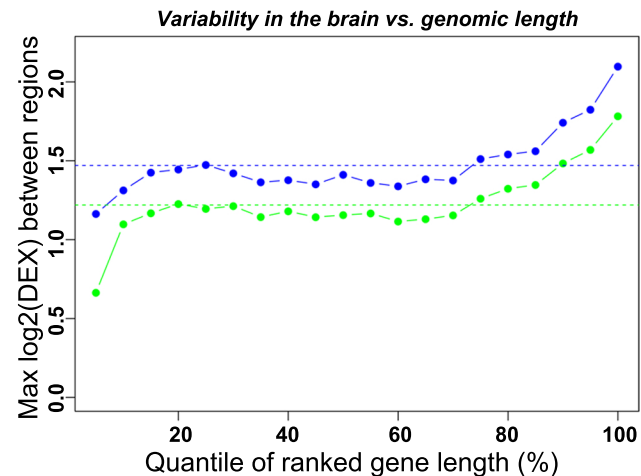

Supplement: Supplementary file 6 — Additional file 6: Gene expression reproducibility is dependent on transcript length. There is a strong linear relationship between gene length—defined based both on the average transcript length for all RefSeq isoforms of the gene as well as for the number of base pairs spanned in the genome—and reproducibility for genes. This is in part because large genes tend to show higher variability in the brain compared with smaller genes. (PDF 6 MB) [file 12864_2013_7016_MOESM6_ESM.pdf]

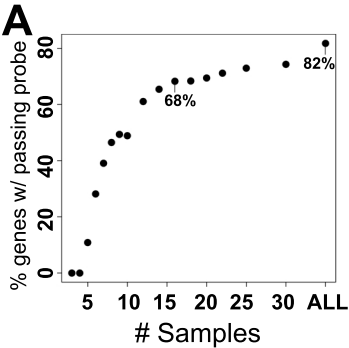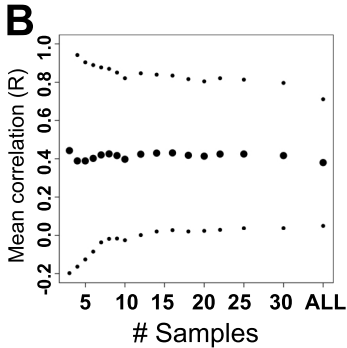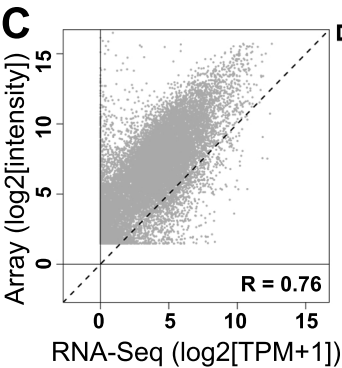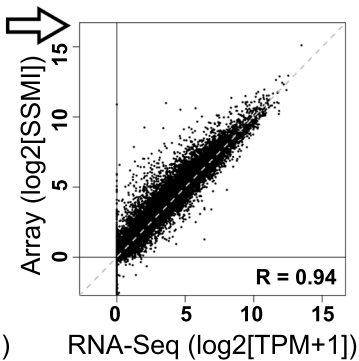

Supplement: Supplementary file 7 — Additional file 7: Microarray quality control and scaling can be accurately done with 16 samples. The percent of passing probes rapidly improves when using a small number of matched samples, and starts leveling out at around 16 samples. Excellent between-method correlation and improvements in scaling are also seen with 16 samples or fewer. (PDF 3 MB) [file 12864_2013_7016_MOESM7_ESM.pdf]
