# Supplementary material for: Improving reliability and absolute quantification of human brain microarray data by filtering and scaling probes using RNA-Seq
Source: BMC Genomics. 2014 Feb 24;15(1):154. doi: 10.1186/1471-2164-15-154 (PMC4007560; doi:10.1186/1471-2164-15-154)
Supplement: Supplementary file 3 — Additional file 3: Supplementary methods and supplementary figure legends. This file contains the supplementary methods for the manuscript followed by supplementary figure legends corresponding to Additional files 2, 4, 5, 6 and 7. (DOCX 28 KB) [file 12864_2013_7016_MOESM3_ESM.docx]

**Table of Contents**

Supplementary Methods: *Page 1*

Supplementary References: *Page 5*

Supplementary Figure Legends: *Page 5*

**---------------------------------------------------------------------------------------------------------------------**

**Supplementary Methods**

*Detailed sample information including brain regions assayed*

All samples used in this analysis are also available as part of the adult human component of the Allen Brain Atlas, as described previously [1, 2] and in the main text. Region names and abbreviations are identical to those used in the ontology, which is accessible at the Allen Institute data portal ([www.brain-map.org](http://www.brain-map.org)). A total of 231 unique macrodissected samples from 29 matched neocortical and non-neocortical regions in two brains were processed. With the exception of the inferior bank of the angular gyrus (Ang-i), which had one failed sample in the left hemisphere of brain 1, each brain region was analyzed in eight independent samples, spanning both hemispheres of both brains, with two independent sampling sites per hemisphere (treated as biological replicates). These regions included:

- Frontal cortex
  - GRe (gyrus rectus)
  - orIFG (inferior frontal gyrus, orbital part)
  - OrbGyri (including LOrG, MOrG, and POrG; lateral, medial, and posterior orbital gyrus)
  - MFG (including MFG-s and MFG-i; superior and inferior banks of the middle frontal gyrus)
  - PCLa-s (paracentral lobule, anterior part, superior bank of gyrus)
  - PCLa-i (paracentral lobule, anterior part, inferior bank of gyrus)
  - PrG (including PrG-prc, PrG-sl, and PrG-il; bank of precentral sulcus, and superior lateral and inferior lateral aspects of precentral gyrus)
  - SFG-m (medial bank of superior frontal gyrus)
  - SFG-l (lateral bank of superior frontal gyrus)
- Parietal cortex
  - ANG-s (superior bank of the angular gyrus) in parietal cortex
  - ANG-i (inferior bank of the angular gyrus) in parietal cortex
  - SMG-i (inferior bank of the supramarginal gyrus) in parietal cortex
  - POG-cs (bank of central sulcus in postcentral gyrus) in parietal cortex
  - POG-l (including POG-sl and POG-il; superior lateral and inferior lateral aspects of postcentral gyrus) in parietal cortex
  - Pcu (including Pcu-i and Pcu-s; superior lateral and inferior lateral banks of precuneus) in parietal cortex
  - SPL (including SPL-i and SPL-s; superior and inferior banks of the supraparietal lobule)
- Temporal cortex
  - FuG-its (fusiform gyrus, bank of the its)
  - ITG (including ITG-its, ITG-mts, and ITG-l; bank of the its and mts and lateral bank in the inferior temporal gyrus)
  - MTG (including MTG-s and MTG-i; superior and inferior banks of the middle temporal gyrus)
  - STG-i (inferior bank of the superior temporal gyrus)
- Occipital cortex
  - pest_V2 (Cun-pest and LiG-pest; peristriate cortex in cuneus and lingual gyrus)
  - str_V1 (Cun-str and LiG-str; striate cortex in cuneus and lingual gyrus)
- Non-neocortical regions
  - CgC (including CgGf-i, CgGf-s, CgGp-i, and CgGp-s; inferior and superior banks of the frontal and parietal parts in the cingulate gyrus)
  - Insula (including LIG and SIG; long and short insular gyri)
  - PHG (including PHG-l and PHG-cos; lateral bank and bank of the cos in the parahippocampal gyrus)
  - GP (including GPe and GPi; external and internal segments of the globus pallidus)
  - Putamen
  - Caudate (HCd; head of the caudate nucleus)
  - CbCx (including He-Crus I&II, He-VI&VIIA, and PV-IV&VIIB&IX; several areas of the hemispheres and paravermis in the cerebellum)

Within each region, samples for sequencing were initially chosen to minimize the overall transcriptional distance (defined as 1 minus the Pearson correlation) based on the microarray data. Only macrodissected samples were considered for RNA-Seq, because these were the only samples with enough RNA leftover from the initial atlasing project to re-run using RNA-Seq.

*Details on preprocessing of RNA-Seq data*

Sequences were aligned to the genome using RNA-Seq by Expectation-Maximization (RSEM) [3] with the following function call:

*rsem-calculate-expression --bowtie-chunkmbs 256 --output-genome-bam --bowtie-e 500 --bowtie-m 100 --paired-end $readfile $readfile2 $indexfile $outname*

where $readfile and $readfile2 are the paired-end FASTQ files for each sample, $indexfile is the gtf file corresponding to the reference human genome (GRCh37/hg19), and $outname indicates the folder for output files. Aligned reads were then processed as described in the text to generate TPM values. Gene length is defined based both on the average transcript length for all RefSeq isoforms of the gene, as well as for the number of base pairs spanned in the genome from the first to the last base pair in the gene.

*Directed differentiation of H1 & H9 cells to cortical neurons*

H1 and H9 hESC were purchased from Wicell and maintained in on matrigel and in mTeSR-1 according to standard practices. Only low passage (<30) cell are used in differentiation experiments. In brief hESC are differentiated to cortical precursors in 24 well plates for 12 days using DMEM/F12 supplemented with SB431542, LDN193189 and Cyclopamine. After 12 days the cells are replated 2 times for 7 days in DMEM/F12 supplemented with bFGF and EGF and differentiated for an additional 4 weeks in DMEM/F12 supplemented with N2/B27 and neurotrophic factors (cyclic AMP, BDNF, GDNF and NT-3).

*Processing of RNA from hESCs*

RNA was generated by lysing H1 & H9 hESC or their day 6, 12, 19, 26 and 54 day cortical neuronal progeny in RNAeasy buffer (Qiagen) and generating the RNA according the standard RNAeasy protocol. RNA was quantified by analysis on Bioanalyser using standard procedures. All final RNA had RIN values of greater than 9. RNA from a total of 16 samples was stored at -80C until all samples were collected and was then sent out to the vendors for processing. Covance processed data for microarray, while or Expression Analysis processed data for RNA-Seq. This is in contrast to the data from brain, which was processed by Cogenics for arrays and by Covance for RNA-Seq.

*Specific metrics for reproducibility involving Pearson correlation*

Pearson correlations (R) were calculated in several contexts as measures of consistency or reproducibility: across genes, we (I) correlated absolute gene expression levels between brains or between methods, and (II) correlated measures of differential expression between brains or between methods; and for each gene, we (III) correlated average expression in each region between brains, and (IV) correlated average expression in each sample between methods. Differential expression was measured in two ways. In the first method, as a global measure of biological or technical reproducibility 100,000 genes and corresponding pairs of non-neocortical regions were randomly chosen, the fold change between these two regions for that gene was estimated, and the correlation was calculated (II above). In the second method, a p-value for each gene was calculated by running ANOVA (using the aov function in R [4]) across either the 7 non-neocortical or 22 neocortical regions. To assess biological reproducibility of gene subsets, genes were sorted based on average expression level or genomic distance between start and stop codon, binned into 20 groups, and then the average and standard deviation of the across-region correlations (III above) were calculated.

*Other approaches for finding scaling parameters*

To calculate sequencing scaled microarray intensities (SSMIs), we identified the 5^th^ and 95^th^ quantile of expression for each microarray probe (using intensity) and for the corresponding RNA-Seq gene (using TPM), and then linearly scaled the microarray intensities so that these values would align with TPMs. We performed this scaling using only samples from brain 2, and reserved brain 1 as an independent test set. A few other scaling methods were also tested as potential methods for scaling, but turned out to be problematic. We first considered linear (least squares) regression, but found that the resulting slope was often too shallow and in some cases was found to be negative, which is not experimentally possible. We next considered orthogonal (total least squares) regression, which minimizes the orthogonal distance to the line of best fit. Scaling using this strategy significantly improved the between-method correlations of many genes relative to unscaled data, while showing fewer of the drawbacks than linear regression. Lastly, we considered and ultimately decided on the quantile method described in the text (i.e., scaling to the 5% and 95% quantiles), because it addressed two of the main problems with both regression strategies: 1) this strategy guaranteed a positive linear relationship between RNA-Seq and microarray expression calls and 2) this strategy was more robust to outlier samples since most highly and most lowly expressed samples are ignored. The quantile method also improved the between-method correlation to a greater extent than orthogonal regression (average R = 0.984 vs. 0.971; see **Fig. 5B**).

*Prioritizing samples for reproducing analyses using fewer samples*

Although our quality control metrics and scaling parameters were found using all 116 samples from brain 2, many of these samples had relatively similar expression patterns overall, and therefore added fairly little power to our analysis. In order to prioritize our samples, we sought to include the N samples with the most distinct expression patterns overall for each 3<N<30. To do this, we used the following strategy: first, we iteratively removed the sample with the highest average interarray correlation to roughly order samples, and then, using this order as base-line, we choose replicates by alternating between brain regions and choosing the most distinct sample in that region. Specific R code for choosing these samples is included in **Additional File 1**. Note that we are not claiming this is the best method for choosing samples, but it seems to work for us. If one were to further optimize sample selection, the conclusions from this analysis would either stay the same or get better, but would not get worse. Therefore, our results could be considered a lower bound.

*Reproducing the analysis*

R code for reproducing the analysis starting with the normalized microarray data, as well as RSEM TPM values and raw counts is included in **Additional File 1**. This document requires additional data and code files that are all available either at the Allen Institute data portal ([www.brain-map.org](http://www.brain-map.org); under the "Download" tab of the Human Brain Atlas), or as supplemental materials with this manuscript (**Additional File 1**). The only data plot not included in this code is the upper left plot of panel B in **Additional File 2**, which could also be reproduced by downloading the raw microarray files individually and reading them into a matrix in R. The analysis was originally done using a desktop computer (3.07GHz processor, 4GB RAM, Windows 7) and R version 2.15.1, and could be run start to finish in less than two days.

**Supplementary References**

1. Hawrylycz M, Lein E, Guillozet-Bongaarts A, Shen E, Ng L, Miller J, van de Lagemaat L, Smith K, Ebbert A, Riley Z, et al: **An anatomically comprehensive atlas of the adult human brain transcriptome.** *Nature* 2012, **489:**391-399.

2. Shen EH, Overly CC, Jones AR: **The Allen Human Brain Atlas: comprehensive gene expression mapping of the human brain.** *Trends Neurosci* 2012, **35:**711-714.

3. Li B, Ruotti V, Stewart RM, Thomson JA, Dewey CN: **RNA-Seq gene expression estimation with read mapping uncertainty.** *Bioinformatics* 2010, **26:**493-500.

4. Chambers JM, Freeny A, Heiberger RM: **Analysis of variance; designed experiments.** In *Statistical Models in S.* Edited by Chambers JM, Hastie TJ: Wadsworth & Brooks/Cole; 1992

**Supplementary Figure Legends**

**Additional File 2**: Clustering of RNA-Seq samples after TbT normalization shows minimal batch effects. Dendrogram (top) shows samples hierarchically clustered based on the Pearson correlation distance (1-R) between each sample (y-axis). Color bars label samples based on various biological and technical variables. "Ontology color" shows the sample color displayed at the Allen Institute web portal. "Brain region" colors samples based on the region of origin—note how samples from striatum, globus pallidus, and cerebellum cluster by region (left three groups). "Hemisphere" indicates whether the sample came from left (turquoise) or right (blue) hemisphere. We see no differential expression based on hemisphere. "Brain of origin" indicates whether the sample is from brain 1 (turquoise) or brain 2 (blue). Samples cluster perfectly based on brain and region, suggested a systematic bias of expression levels between brains, which we correct using ComBat (see also **Additional File 4**). Colors corresponding to technical variables—"Clip percentage" (how much sequences were trimmed), "Number of clusters" (how many total fragments were there), "Microarray batch" (from the initial atlasing project), "RIN (RNA-quality)", and "RNA-Seq batch" (sequence run)—all appear randomized, suggesting that there are no gross batch effects, issues with the sequencing, or clustering due to RNA quality. Note that several RNA-Seq batches included only samples from a single brain, which entirely explains the grouping of blue and turquoise from the remaining colors.

**Additional File 4**: Normalization improves the quality of RNA-Seq and microarray data. **A**) Box plots of gene expression levels (y-axis) showing the median, 25% and 75% quantiles (edges of boxes), 5% and 95% quantiles (edges of dotted lines), and outliers (circles) for brain 2. Samples are ordered by the ontology, although the order is not important for this plot. Note the better alignment of boxes between samples after Tbt normalization, indicating that the expression levels after normalization are more comparable. Similar plots were found for brain 1 (not shown). **B**) Correlation of differential expression between brains based on microarray intensity (top row) and RNA-Seq TPM values (bottom rows) for raw data (left), normalized data (center; standard method for microarray, Tbt normalization for RNA-Seq), and ComBat normalization with respect to brain (right). Each of 100,000 points shows the log2 fold change of a random gene between two random non-neocortical regions as measured by brain 1 (x-axis) and brain 2 (y-axis). Note the improved correlation (R) after each normalization step. **C**) Multidimensional scaling (MDS) plot of samples showing the first two principal components that explain the most variance (x and y-axes). Rows and columns as in B. Sampled are labeled based on their brain region of origin and color coded based on brain (brain 1 = turquoise, brain 2 = blue). "Sc" is a numerical score which quantifies how well samples from brain 1 cluster with samples from brain 2 (lower is better). Specifically, Sc is the average Euclidean distance between samples from the same region in brain 1 compared with brain 2 divided by the average Euclidean distance between all samples across brains. Note both visually and by the decrease in score how ComBat normalization significantly improves the alignment of samples so that samples from the same region tend to cluster together, regardless of the brain of origin. Furthermore, the microarray and RNA-Seq plots look very similar after ComBat normalization. The y-axis on three plots have been inverted so that cortical samples appear at the bottom left of the plot, which makes no difference mathematically.

**Additional File 5**: Many probes with specifically high microarray intensity do not accurately measure gene expression. **A**) Probes with specifically high expression in microarray (i.e., the red dots in **Fig. 2B**) show poor between-method agreement based on differential expression levels between regions, suggesting they do not appropriately measure expression of their assigned gene. Labeling as in **Fig. 2G**. **B**) These probes show good between-brain agreement, suggesting that they are consistently measuring something and are not showing random high expression levels. Labeling as in **Fig. 2E**. **C**) These probes are more likely than chance to be part of gene families. Gene families are crudely defined as genes with the same first three letters in their gene symbol ("prefix"). One thousand randomly selected groups of 670 genes were chosen, and a histogram of the number of unique prefixes from each group is plotted and compared with the 257 identified for the specifically highly expressed probes (vertical black line). Each bar represents the percent of permutations (y-axis) for which the number of unique prefixes falls in the shown range (x-axis).

**Additional File 6**: Gene expression reproducibility is dependent on transcript length. Gene length is defined based both on the average transcript length for all RefSeq isoforms of the gene (**A**) as well as for the number of base pairs spanned in the genome from the first to the last base pair in the gene (**B**). Top row) There is a strong linear relationship between gene length and reproducibility for genes. Genes were sorted from smallest to largest and divided into 20 bins based on size, which each represent 5% of array genes (x-axis). Each point shows the average between-brain correlation (as in **Fig. 3A**) for all genes in that bin (y-axis), as measured by microarray (blue) and RNA-Seq (green). Horizontal dotted lines show the average correlation across all genes for that method. Bottom row) Large genes tend to show higher variability in the brain compared with smaller genes. Variability is defined as the maximum differential expression between any two pairs of regions for a given gene, and is shown in log2 space (y-axis). Bins as in the top row. While this effect is seen using both genomic and transcriptomic length, genes spanning a large distance on the genome are particularly likely to be differentially expressed across the brain. This result explains the relationship between gene length and reproducibility seen with microarray, at least in part.

**Additional File 7**: Quality control metrics and scaling parameters are relatively accurate when using only 16 samples. **A**) The percentage of genes with at least one probe passing quality control metrics (y-axis; as described in the text) is highly dependent on the number of samples included in the analysis (x-axis) when fewer than 16 samples are used. Only slight increases in the number of passing probes are seen between 16 samples (68%) and all 116 samples (82%). Separate analyses were performed using between 3-30 carefully selected samples, and compared against the original analysis. Details about how samples are chosen can be found in the **Supplementary Methods**. **B**) The average probe-level correlation of gene expression between brains (y-axis; as shown in **Fig. 4A**) is independent of the number of samples included in the analysis. Average correlation (large dots) +/- one standard deviation (small dots) is shown for the analyses generated in **A**. After scaling using scaling parameters generated by the 16-sample analysis (black dots), all 115 samples in brain 1 show markedly improved between-method correlation of absolute expression levels compared with before (grey dots). The sample shown in **Fig. 5C** is shown here. Labeling as in **Fig. 5C**. Note that the unscaled plots here and in **Fig. 5C** look slightly different because only the best passing probe for each gene in shown.
