# Supplementary material for: Improving reliability and absolute quantification of human brain microarray data by filtering and scaling probes using RNA-Seq
Source: BMC Genomics. 2014 Feb 24;15(1):154. doi: 10.1186/1471-2164-15-154 (PMC4007560; doi:10.1186/1471-2164-15-154)
Supplement: Supplementary file 4 — Additional file 4: Normalization improves the quality of RNA-Seq and microarray data. Several plots showing that RNA-Seq data becomes progressively more consistent and reproducible after TbT normalization, which scales for the total reads, and ComBat normalization, which removes the systematic bias between brains. Microarray data likewise improved. (PDF 18 MB) [file 12864_2013_7016_MOESM4_ESM.pdf]

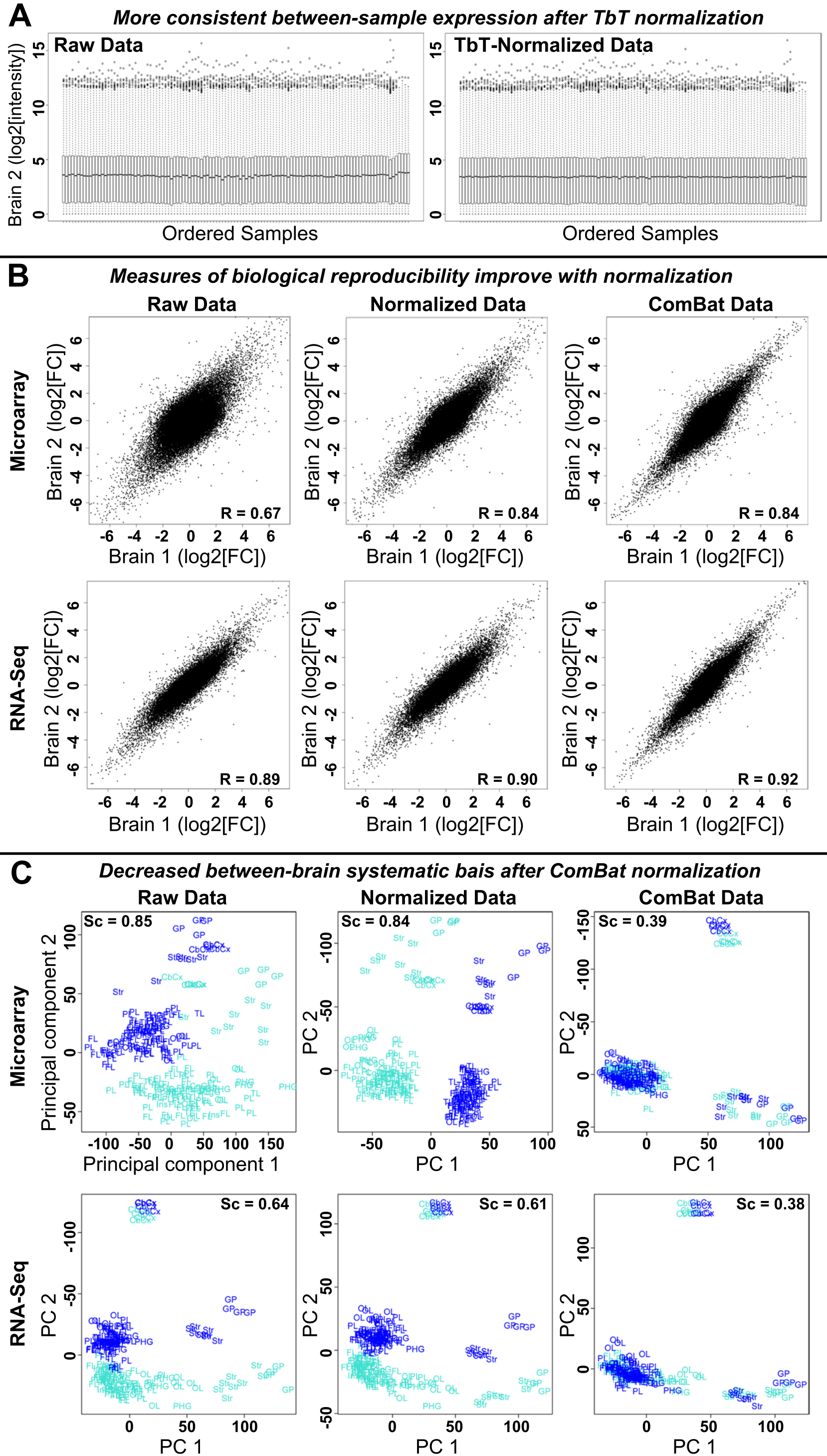
A
More consistent between-sample expression after TbT normalization
Raw Data
TbT-Normalized Data
Brain 2 (log2[intensity])
Ordered Samples
Ordered Samples
B
Measures of biological reproducibility improve with normalization
Raw Data
Normalized Data
ComBat Data
Microarray
RNA-Seq
Brain 2 (log2[FC])
Brain 1 (log2[FC])
R = 0.67
R = 0.84
R = 0.84
R = 0.89
R = 0.90
R = 0.92
C
Decreased between-brain systematic bais after ComBat normalization
Raw Data
Normalized Data
ComBat Data
Microarray
RNA-Seq
Principal component 2
Principal component 1
PC 2
PC 1
Sc = 0.85
Sc = 0.84
Sc = 0.39
Sc = 0.64
Sc = 0.61
Sc = 0.38
