# Supplementary material for: Improving reliability and absolute quantification of human brain microarray data by filtering and scaling probes using RNA-Seq
Source: BMC Genomics. 2014 Feb 24;15(1):154. doi: 10.1186/1471-2164-15-154 (PMC4007560; doi:10.1186/1471-2164-15-154)
Supplement: Supplementary file 1 — Additional file 1: Code and data to reproduce analysis. This zip file contains two code documents and several supporting data files that are required, along with data from the Allen Brain Atlas data portal (http://www.brain-map.org) to reproduce nearly all figures and statistics presented in this manuscript. (ZIP 11 MB) [file 12864_2013_7016_MOESM1_ESM.zip › Additional_File_1/RNA_Seq_Microarray_Code_SingleDocument_1_27_14.doc]

```
## --------------------------------------------------------------------------------------------- ##
##                           Code document associated with the paper:                            ##
##      "Improving reliability and absolute quantification of human brain microarray data by     ##
##                          filtering and scaling probes using RNA-seq"                          ##
## --------------------------------------------------------------------------------------------- ##


## General comments on computing power

# This code can be run on a desktop computer (3GHz hard drive, 4GB RAM) in a day or two.  Depending on your RAM, you may need to include steps for deleting unnecessary files from the R project (using the "rm" command) and making sure to occasionally run the "collectGarbage" command.  If you have any issues, please let me know:  Jeremy Miller – jeremym@alleninstitute.org


## Installing R

# Before starting you will need to install a recent version of R (R 2.15.3 for windows can be installed from this link: http://cran.r-project.org/bin/windows/base/old/2.15.3/R-2.15.3-win.exe).  I think this code works with R 3.0, but am not 100% sure.  There are a few libraries required to run this code.  If you don't have them installed, you will get an error that will tell you which library you need to install.  To install libraries, you will need to use one of two stragies, depending on the library:

# 1. Type the following, replacing LIBRARY with the library name in "quotes"
# (this IS case sensitive):
install.packages(LIBRARY)

# 2. Type the following two lines, replacing LIBRARY with the library name in "quotes"
#(this IS case sensitive):
source("http://bioconductor.org/biocLite.R")
biocLite(LIBRARY)

# If none of the above works, your packages might be out of date.  To address this, type the following and say "Yes" for all the packages:
update.packages()


## Downloading and storing the data

# First you need to choose a working directory.  This is where you will store all of your data and where you will run the code from.

# Download and unzip additional file 1 from "Improving reliability and absolute quantification of human brain microarray data by filtering and scaling probes using RNA-seq".  Since you are reading this, you probably have already unzipped the file!  Move the four files into a subfolder of your working directory called "dataFilesForAnalysis" (without the quotes).

# Download and unzip "Complete normalized microarray datasets" for brains 1 and 2 (H0351.2001 and H0351.2002) from http://human.brain-map.org/static/download.  Move the files in these data sets into subfolders of your working directory called "H2001_array" and "H2002_array", respectively.

# Download and unzip "RNA-Sequencing datasets" for brains 1 and 2 (H0351.2001 and H0351.2002) from the same location as well.  Move the files in these data sets into subfolders of your working directory called "H2001_seq" and "H2002_seq", respectively.

## YOU ARE NOW READY TO BEGIN! ##


## Start R and set the current working directory
# To set the working directory, replace the line in green with your working directory:

wd = "C:/Users/jeremym/Desktop/ALL ABI STUFF/RNA-seq stuff/AnalysisStartToFinish_fromWebsite"
setwd(wd)


## Read in and format the data

# This step is for reading in the raw microarray data.  These are the only data that are not available in an easy-to-access format.  If you want to get the raw data, go to http://human.brain-map.org/static/download and follow the directions for downloading the raw data.  As these data are only used for one panel in a supplemental figure, I would suggest not worrying about this step.

dataRawA = read.csv("microarrayLog2Intensities_raw.csv",row.names=1)

## Read in the data from the paper

setwd(paste(wd,"/dataFilesForAnalysis",sep=""))
source("functionsForRNAseqMicroarrayComparison.r")      # Function with all additional files
datESCR = read.csv("RNAseqLog2TPMplus1_ESC.csv",row.names=1)            # These data will be
datESCA = read.csv("microarrayLog2Intensities_ESC.csv",row.names=1)     # used for Figure 6.

## Read in and subset the RNA-seq data

# BRAIN 1
setwd(paste(wd,"/H2001_seq",sep=""))
genesSeq   = read.csv("Genes.csv",row.names=1)
sampleSeq1 = read.csv("SampleAnnot.csv",row.names=1)
datSeq1    = read.csv("RNAseqTPM.csv",row.names=1,header=FALSE)
countSeq1  = read.csv("RNAseqCounts.csv",row.names=1,header=FALSE)

# BRAIN 2
setwd(paste(wd,"/H2002_seq",sep=""))
sampleSeq2 = read.csv("SampleAnnot.csv",row.names=1)
datSeq2    = read.csv("RNAseqTPM.csv",row.names=1,header=FALSE)
countSeq2  = read.csv("RNAseqCounts.csv",row.names=1,header=FALSE)

sampleSeq  = rbind(sampleSeq1,sampleSeq2)              # Combine the data from the two brains
datSeq     = cbind(datSeq1,datSeq2)
countSeq   = cbind(countSeq1,countSeq2)

kpSeq      = sampleSeq$replicate_sample=="No"          # (For the paper, we omitted replicates)
sampleSeq  = sampleSeq[kpSeq,]
datSeq     = datSeq[,kpSeq]
countSeq   = countSeq[,kpSeq]
sampleSeq$long_name = rownames(sampleSeq)
samps <- rownames(sampleSeq) <- sampleSeq$sample_name  # samps will be used later for sample names
wellid     = as.character(sampleSeq$well_id)           # to match data with microarray

library(WGCNA)
rm(sampleSeq1,sampleSeq2,datSeq1,datSeq2,countSeq1,countSeq2)
collectGarbage()  # This function cleans up the workspace.

## Read in and subset the microarray data

# BRAIN 1
setwd(paste(wd,"/H2001_array",sep=""))
probesArray  = read.csv("Probes.csv",row.names=2)
sampleArray1 = read.csv("SampleAnnot.csv",row.names=3)
kpArray1     = is.element(rownames(sampleArray1),wellid)
datArray1    = read.csv("MicroarrayExpression.csv",row.names=1,header=FALSE)
PAcall1      = read.csv("PACall.csv",row.names=1,header=FALSE)
sampleArray1 = sampleArray1[kpArray1,]
datArray1    = datArray1[,kpArray1]
PAcall1      = PAcall1[,kpArray1]

# BRAIN 2
setwd(paste(wd,"/H2002_array",sep=""))
sampleArray2 = read.csv("SampleAnnot.csv",row.names=3)
kpArray2     = is.element(rownames(sampleArray2),wellid)
datArray2    = read.csv("MicroarrayExpression.csv",row.names=1,header=FALSE)
PAcall2      = read.csv("PACall.csv",row.names=1,header=FALSE)
sampleArray2 = sampleArray2[kpArray2,]
datArray2    = datArray2[,kpArray2]
PAcall2      = PAcall2[,kpArray2]

sampleArray  = rbind(sampleArray1,sampleArray2)        # Combine the data from the two brains
datArray     = cbind(datArray1,datArray2)
PAcall       = cbind(PAcall1,PAcall2)
orderSeq     = match(wellid,rownames(sampleArray))     # Reorder array data to agree with RNA-seq
sampleArray  = sampleArray[orderSeq,]
datArray     = datArray[,orderSeq]
PAcall       = PAcall[,orderSeq]
colnames(datArray) <- colnames(PAcall) <- rownames(sampleArray) <- samps
rownames(datArray) <- rownames(PAcall) <- rownames(probesArray)

rm(sampleArray1,sampleArray2,datArray1,datArray2,PAcall1,PAcall2)
collectGarbage()  # This function cleans up the workspace.

## DATA READ-IN COMPLETE! ##


## Reformat and rename some of the variables for use in the analysis
# ==> Note that for the remainder of the analysis variables with an "A" (and in some cases "E")
#     correspond to microArray and variables with an "R" correspond to RNA-seq.

dataA      = datArray                        # Note that all of the comparisons are in log2 space
# dataRawA   = dataA
# ==> I would recommend running this line so that the code will run properly.  Just note that if
#     you do, the dataRawA variable will NOT be showing raw data, but rather normalized data.

probesA    = rownames(probesArray)
dataRawA   = dataRawA[probesA,]
genesA     = as.character(probesArray$gene_symbol)
numPAcallA = rowSums(PAcall)                       # In how many samples is the probe "present"?
names(genesA) <- names(numPAcallA) <- probesA

datExprR   = log2(1000000*datSeq + 1)
# ==> This is a critical step.  We multiply by 1000000 because TPM is scaled so that the sum of
#     the gene values per sample is 1, but we want transcripts per million.  We add one so that
#     when we convert to log2 space, we don't end up with negative infinity values.  This also
#     ensures that a TPM of 1 and a log2-transformed TPM of 1 are the same value.  This could
#     arguably create linearity issues for very low transcripts, but we have found that the
#     results are better if we add one here.

genesR     = rownames(datExprR)
sampleInfo = cbind(sampleSeq,sampleArray)
sampleInfo$toplevel  = sampleInfo$main_structure  # For consistency with later code
sampleInfo$subregion = sampleInfo$sub_structure

setwd(wd)
trim.leading <- function (x)  sub("^\\s+", "", x)
collectGarbage()

## DATA PREPARATION COMPLETE! ##
## For most of the analysis, we only consider a single probe for each microarray gene, and only for the genes which are commonly measured using RNA-Seq and microarray.

# ==> We have previously found that, without any additional data to inform our method, choosing the
#     probe with the highest expression is better than several other probe-selection strategies.
#     These are the default parameters in the "collapseRows" function, and what we use here.

datExprA  = collapseRows(dataA,genesA,probesA)
datExprE  = datExprA[[1]]
probesKpA = as.character(datExprA[[2]][,2])
genesKpA  = genesA[probesKpA]
names(probesKpA) = genesKpA
collectGarbage()

## Find the subset of genes available via both methods & only consider these genes in the analysis

genes     = sort(intersect(genesKpA,genesR))
length(genes)  # [1] 18761                     # Number of genes in common across methods.
probes    = probesKpA[genes]
datExprE  = datExprE[genes,]
datExprR  = datExprR[genes,]
datCountR = countSeq[genes,]
datRawE   = dataRawA[probes,]
rownames(datRawE) = genes
collectGarbage()


## Search for and (potentially) remove outliers

#  ==> The microarray part of this section is not in the paper, so don't worry about not
#      having actual raw data here.

colnames(datExprR) = colnames(datExprE)
cmdEt    = cmdscale(dist(t(datRawE)),2);    cmdRt = cmdscale(dist(t(datExprR)),2)
s        = sampleInfo;                      text  = as.character(s[,6]);
cols     = as.character(s[,5])

pdf("MDS_samples_for_each_method_rawF.pdf", width=11,height=6);  par(mfrow=c(1,2))
plot(cmdEt,xlab="PC 1", ylab="PC 2", main="Array",col="white")
text(cmdEt[,1],cmdEt[,2],text,col=cols)
plot(cmdRt[,1],-cmdRt[,2],xlab="PC 1", ylab="-PC 2", main="RSEM",col="white")
text(cmdRt[,1],-cmdRt[,2],text,col=cols)
dev.off();
```

```
# Nothing looks obviously wrong from this clustering in terms of outliers, although there do appear to be two clusters of everything.  Raw array data also appears to be much messier than raw RNA-Seq data, which is not surprising.

sampLong  = paste(rownames(s),s[,6],s[,7],s[,8],sep="_")
IAC_E     = cor(datRawE,use="p");        IAC_R     = cor(datExprR,use="p");
rownames(IAC_E) <- colnames(IAC_E) <- rownames(IAC_R) <- colnames(IAC_R) <- sampLong
cluster_E = hclust(as.dist(1-IAC_E));     cluster_R = hclust(as.dist(1-IAC_R))
colVector = cbind(cols,labels2colors(s[,6]),labels2colors(s[,8]),labels2colors(s[,9]),
   numbers2colors(as.numeric(s[,11])),numbers2colors(as.numeric(s[,10])),labels2colors(s[,4]),
   numbers2colors(as.numeric(s[,12])),labels2colors(s[,13]))
colnames(colVector) = c("Region","Toplevel","Hemisphere","Brain","Clip %","# Clusts.","ArrayBatch",
   "RIN","RunID_Seq")

pdf("IAC_each_method_rawF.pdf",width=35,height=15);
plotDendroAndColors(cluster_E,colVector,main="Array")
plotDendroAndColors(cluster_R,colVector,main="RNA-Seq")
dev.off()
```

\# In the raw RSEM data, there is a large brain effect. Note that the array data is already normalized, but that there is still an even larger brain effect. In array there also appear to be batch effects that are smaller than the biological effects. In RNA-Seq there also may be effects of RunID (i.e., batch), but they are smaller. Both RNA-Seq and microarray cluster GP, striatum, and cerebellum distinctly from the rest.

*# I don't see any evidence from the RSEM data to remove any samples. Samples were chosen for this pilot study after removing outlier samples from microarray, and hence there are no microarray outlier samples.*

```
###################################################################################################
## RNA-SEQ NORMALIZATION STRATEGIES (i.e., for aligning samples and removing brain bias)

## First perform the TbT strategy from Kadota et al 2012 ######
# ==> Define the two comparisons groups as cortex vs. any of the other regions, so that TbT normalization can omit genes differentially expressed between these groups.
# Note that this section takes a long time to run (a few hours)

cortexType = is.element(s$main_structure,c("TL","PL","OL","FL"))

set.seed(111)                               # To ensure that all runs get the exact same result...
datExprRn  = normalizeByTbT((2^datExprR)-1, cortexType)
c(mean(2^datExprR-1),mean(datExprRn))       # TbT normalization screws up the scale!  Scale to
# [1]  47.91523 366.00763                   # original TPM values, then convert back to log2 space
scaleTbT   = (mean(2^datExprR-1))/mean(datExprRn)
datExprRn  = log2(datExprRn*scaleTbT+1)
save(datExprRn,file="datExprRn.RData")     # To save this file, uncomment this line.


## How do the MDS plots before vs. after normalization look

cmdRn = cmdscale(dist(t(datExprRn)),2);    cmdEn = cmdscale(dist(t(datExprE)),2);
                                           # (The array data has already been normalized.)
pdf("MDS_samples_for_each_method_TbT_F.pdf", width=17,height=9);  par(mfrow=c(2,4))
plot(cmdEt,xlab="PC 1", ylab="PC 2", main="Array - Raw",col="white")
text(cmdEt[,1],cmdEt[,2],text,col=cols)
plot(cmdEn,xlab="PC 1", ylab="PC 2", main="Array - Norm",col="white")
text(cmdEn[,1],cmdEn[,2],text,col=cols)
plot(cmdRt,xlab="PC 1", ylab="PC 2", main="RSEM - Raw",col="white")
text(cmdRt[,1],cmdRt[,2],text,col=cols)
plot(cmdRn,xlab="PC 1", ylab="PC 2", main="RSEM – TbT Norm.",col="white")
text(cmdRn[,1],cmdRn[,2],text,col=cols)
plot(cmdEt,xlab="PC 1", ylab="PC 2", main="Array - Raw",col="white")
text(cmdEt[,1],cmdEt[,2],text,col=labels2colors(s[,9]))
plot(cmdEn,xlab="PC 1", ylab="PC 2", main="Array - Norm",col="white")
text(cmdEn[,1],cmdEn[,2],text,col=labels2colors(s[,9]))
plot(cmdRt,xlab="PC 1", ylab="PC 2", main="RSEM - Raw",col="white")
text(cmdRt[,1],cmdRt[,2],text,col=labels2colors(s[,9]))
plot(cmdRn,xlab="PC 1", ylab="PC 2", main="RSEM – TbT Norm.",col="white")
text(cmdRn[,1],cmdRn[,2],text,col=labels2colors(s[,9]))
dev.off();
```

```
# Based on these plots, normalization appears to make very little difference in log2 space by this measure for RNA-Seq.  In microarray, it improves the results (at least within each brain), as shown previously. However, note that both data sets have some pretty major differences between brains using this measure.  This will be addressed later using ComBat.  I should note that the normalizations for microarray and RNA-Seq are NOT THE SAME.  Finally, we note that reflections along the x and y axes are not particularly meaningful for principal components.

## Plot some box plots of the TbT normalized and unnormalized data to test the results

whichBrain = as.integer(s[,9])
w1 = whichBrain==1;   w2 = whichBrain==2
pdf("boxplotsRSEM_F.pdf",height=16,width=32); par(mfrow=c(2,2))
boxplot(as.data.frame(datExprR[,w1]),las=2,main="Raw, Brain 1")
boxplot(as.data.frame(datExprR[,w2]),las=2,main="Raw, Brain 2")
boxplot(as.data.frame(datExprRn[,w1]),las=2,main="Normalized, Brain 1")
boxplot(as.data.frame(datExprRn[,w2]),las=2,main="Normalized, Brain 2")
dev.off()
```

```
# TbT normalization makes a big difference with RSEM.  This makes sense because these are basically the actual counts rather than the RPKM values.  I.e., it has not already been normalized.

## Summary statistic for the box plots

c(sd(apply(datExprR,2,median)), sd(apply(datExprRn,2,median)))
# [1] 0.15386868 0.05011326
# There is a 3-fold decrease in standard deviation of median gene expression levels between samples after applying TbT normalization.

## Replot the dendrogram of samples as above

IAC_Rn     = cor(datExprRn,use="p");    rownames(IAC_Rn) <- colnames(IAC_Rn) <- sampLong
cluster_Rn = hclust(as.dist(1-IAC_Rn))
pdf("IAC_RSEM_normalizedF.pdf",width=35,height=15);
plotDendroAndColors(cluster_Rn,colVector,main="RNA-Seq")
dev.off()
```

```
# This is very similar to the plot above (although a few minor branches have been swapped)

###################################################################################################
## Plot expression levels and differential expression between brains 1 and 2

r <- function(x) return(rowMeans(x))
kpPres    = !(r(datExprR)==0);                           # Almost 99% of genes are present in at
sum(kpPres)/length(kpPres)               # [1] 0.9868344 #  least 1 sample as measured by RNA-Seq.
sum(numPAcallA[probes]>0)/length(kpPres) # [1] 0.9779862 # Likewise, ~98% are present via array.
# Note: we need to remove all genes absent in all samples via RNA-Seq to avoid divide-by-0 errors.
datR1avg  = t(findFromGroups(t(datExprR[kpPres,w1]),as.character(sampleInfo$main_structure[w1])))
datR2avg  = t(findFromGroups(t(datExprR[kpPres,w2]),as.character(sampleInfo$main_structure[w2])))
datR1avgN = t(findFromGroups(t(datExprRn[kpPres,w1]),as.character(sampleInfo$main_structure[w1])))
datR2avgN = t(findFromGroups(t(datExprRn[kpPres,w2]),as.character(sampleInfo$main_structure[w2])))
datE1avg  = t(findFromGroups(t(datRawE[kpPres,w1]),as.character(sampleInfo$main_structure[w1])))
datE2avg  = t(findFromGroups(t(datRawE[kpPres,w2]),as.character(sampleInfo$main_structure[w2])))
datE1avgN = t(findFromGroups(t(datExprE[kpPres,w1]),as.character(sampleInfo$main_structure[w1])))
datE2avgN = t(findFromGroups(t(datExprE[kpPres,w2]),as.character(sampleInfo$main_structure[w2])))
subs      = rownames(datR1avg)

## Plot present/absent calls for different numbers of samples. (BELOW LEFT)

nThresh = c(1+c(0,0.5,2.5,5,7.5,9.5,10)*23);  aOnly <- rOnly <- both <- rep(0,length(nThresh));
for (n in 1:length(nThresh)){
  isR     = rowSums(datExprR>0)>=nThresh[n];       isA = numPAcallA[probes]>=nThresh[n];
  both[n] = sum(isA&isR);   aOnly[n] = sum(isA&(!isR));    rOnly[n] = sum(isR&(!isA))
}
total = both+aOnly+rOnly;      datPlot = 100*rbind(both/total,rOnly/total,aOnly/total)
colnames(datPlot) = round(nThresh/2.31);   rownames(datPlot) = c("Common","RNA-Seq","Array")
barplot(datPlot,beside=FALSE,ylab="% present genes",xlab="% samples present",ylim=c(0,100),las=2,
 col=c("#000000","#888888","#DDDDDD"))

# (BELOW LEFT) There are more genes present at every threshold via RNA-Seq than via microarray

## How many total DEX genes are found with each method? (BELOW RIGHT)

pdf("GenesExpressedInHowManySamples.pdf",width=2.8,height=6)
datPlot = rbind(rOnly+both,aOnly+both);    kp = c(2,4,6)
colnames(datPlot) = round(nThresh/2.31);   rownames(datPlot) = c("RNA-Seq","Array")
barplot(100*datPlot[,kp]/dim(datExprR)[1],beside=TRUE,ylab="% genes present",xlab="% of samples",
 ylim=c(0,100),col=c("#888888","#DDDDDD"));   abline(h=10*(6:10),lty="dotted");
for (i in 1:3){ y = 100*both[kp][i]/length(isR); x = i*3-2.25; segments(x,y,x+2.5,y,lwd=3) }
dev.off()

# (BELOW RIGHT) Nearly all genes are present in at least 1 sample.  Using a smaller threshold, ~90% of genes are present (75% ubiquitous) via RNA-Seq, and ~80% are present (~60% ubiq.) via array
```

```
## WHICH genes are present in RNA-Seq and array only in >10% of samples?
# ==> This information will be important later in the analysis

isR = rowSums(datExprR>0)>=24;    isA = numPAcallA[probes]>=24;
onlyRSEM  = genes[isR&(!isA)];    onlyArray = genes[isA&(!isR)]


## Peform all comparisons for the raw and normalized data.

# ==> Note that many of the analyses are wrapped into the function "summaryStatsAndPlots".
#     These plots and statistics are all described in the manuscript.  The important thing
#     to note here is that each function call creates a subfolder (named the fifth parameter),
#     within which several plots and a statistics file are generated.  The statistics typically
#     include numbers in the titles of the various plots.  This function is somewhat annotated
#     and could be viewed from the file "functionsForRNAseqMicroarrayComparison.r".  This
#     function typically takes around 10-15 minutes to run.
# ==> Note that if for some reason this function crashes, you will need to reset the working
#     directory by typing setwd(wd) before doing anything else.

datRawE2 = datRawE;  rownames(datRawE2) = probes      # For this function, need rows to be probes.
summaryStatsAndPlots(datRawE2, datExprR, probes, genes, "rawPlots")
datExprE2 = datExprE;  rownames(datExprE2) = probes   # For this function, need rows to be probes.
summaryStatsAndPlots(datExprE2, datExprRn, probes, genes, "normalizedPlots")

## Plot the average gene expression levels in brains 1 vs. 2 for each method.
```

\# With a few exceptions, expression levels in brains 1 and 2 are VERY close. This is in contrast to the observation that samples from brains 1 and 2 cluster separately. Note that the normalized and unnormalized data look the same based on this plot.

**## Plot the average differential expression fold changes in brains 1 vs. 2 for each method.**

\# Note that (normalized) microarray and RNA-Seq produce nearly identical distributions of differential expression between brain.

```
###################################################################################################
## Run ComBat on the RNA-Seq and array data and see if this improves upon the above normalization.
# Note that ComBat is appropriate in this context because the "batches" are exactly matched (i.e., identical samples in brain 1 and brain 2 were used in the analysis).

toplevel      = as.character(sampleInfo$main_structure) # For later... it is NOT better including this
whichBrain    = as.numeric(sampleInfo$brain)      #   as a covariate.
inf           = cbind(colnames(datExprE),colnames(datExprE),whichBrain)
colnames(inf) = c("Array_name","Sample_name","Batch")
write.table(inf,"info.txt",sep="\t",row.names=FALSE,quote=FALSE)

pdf("ComBat_plots_F.pdf")
write.csv(datExprE[kpPres,],"datEF.csv",row.names=FALSE)
set.seed(123)  # So the results can be exactly reproduced.
datComE=ComBat("datEF.csv","info.txt",type="csv",write=F,par.prior=T,filter=F,skip=0,prior.plots=T)
write.csv(datExprRn[kpPres,],"datRF.csv",row.names=FALSE)
set.seed(321)  # So the results can be exactly reproduced.
datComR=ComBat("datRF.csv","info.txt",type="csv",write=F,par.prior=T,filter=F,skip=0,prior.plots=T)
rownames(datComE) <- rownames(datComR) <- rownames(datExprE)[kpPres]
dev.off()
#            MICROARRAY (LEFT)                              RNA-Seq (RIGHT)
```

```
# save(datComR,datComE,file="ComBat_normalized_data.RData") # Uncomment this line to save file


## Peform all comparisons for the ComBAT data.

datComE2 = datComE;  rownames(datComE2) = probes[kpPres]  # The rows need to be probes.
summaryStatsAndPlots(datComE2, datComR, probes[kpPres], genes[kpPres], "combatPlots")

## Plot the average expression levels and differential expression fold changes in brains 1 vs. 2.
```

```
# We find nearly perfect correlation of mean expression levels between brains using both methods because this constraint is imposed on the data by ComBat.  Of more interest, ComBat makes the differential expression agreement between brains slightly better (in RNA-Seq and possible microarray), which justifies the use of this method in this context.


## Does ComBat even get rid of the batch (aka brain) effects?  Run MDS to find out.

cmdEc = cmdscale(dist(t(datComE)),2);     cmdRc = cmdscale(dist(t(datComR)),2)

pdf("MDS_samples_for_each_method_ComBat_F.pdf", width=15,height=11)
s = sampleInfo; par(mfrow=c(2,3))
plot(cmdEt,xlab="PC 1", ylab="PC 2", main="Array (raw)",col="white")
text(cmdEt[,1],cmdEt[,2],text,col=labels2colors(substr(rownames(s),3,3)),cex=1.5)
plot(cmdEn[,1],-cmdEn[,2],xlab="PC 1", ylab="-PC 2", main="Array (norm.)",col="white")
text(cmdEn[,1],-cmdEn[,2],text,col=labels2colors(substr(rownames(s),3,3)),cex=1.5)
plot(cmdEc[,1],-cmdEc[,2],xlab="PC 1", ylab="-PC 2", main="Array (ComBat)",col="white")
text(cmdEc[,1],-cmdEc[,2],text,col=labels2colors(substr(rownames(s),3,3)),cex=1.5)
plot(cmdRt[,1],-cmdRt[,2],xlab="PC 1", ylab="-PC 2", main="RNA-Seq (raw)",col="white")
text(cmdRt[,1],-cmdRt[,2],text,col=labels2colors(substr(rownames(s),3,3)),cex=1.5)
plot(cmdRn,xlab="PC 1", ylab="PC 2", main="RNA-seq (norm.)",col="white")
text(cmdRn[,1],cmdRn[,2],text,col=labels2colors(substr(rownames(s),3,3)),cex=1.5)
plot(cmdRc,xlab="PC 1", ylab="PC 2", main="RNA-seq (ComBat)",col="white")
text(cmdRc[,1],cmdRc[,2],text,col=labels2colors(substr(rownames(s),3,3)),cex=1.5)
dev.off();
```

```
# (LEFT) ComBat almost entirely removes brain effects, based on this measure (note the overlap between the blue and turqouse text in the right column).  Also note that the array data largely looks like the RSEM data now. (The flip along the y axis is not mathematically meaningful).

## Quantify the differences in the MDS both before and after ComBat normalization
# ==> Define this as the average distance between matching samples in the MDS divided by the average distance between all samples on the MDS

scoreEt = scoreMDS(cmdEt, whichBrain,toplevel)
scoreRt = scoreMDS(cmdRt, whichBrain,toplevel)
scoreEn = scoreMDS(cmdEn, whichBrain,toplevel)
scoreRn = scoreMDS(cmdRn, whichBrain,toplevel)
scoreEc = scoreMDS(cmdEc, whichBrain,toplevel)
scoreRc = scoreMDS(cmdRc, whichBrain,toplevel)

barplot(cbind(c(scoreEt,scoreEn,scoreEc),c(scoreRt,scoreRn,scoreRc)),beside=TRUE,
   names.arg=c("Array","RNA-Seq"))
# ABOVE RIGHT
# Note the significant decrease in within-region distance on the MDS plot after applying ComBat normalization.


## Make some plots showing the general agreement of expression level between methods (brain 2)
# Note that these plots were generated in summaryStatsAndPlots above
#        RAW                  TbT/Standard               ComBat
```

```
# There is VERY good agreement between average expression levels of microarray and RNA-Seq (R~0.78), whether or not we use ComBat normalization.  (Note: while the correlation gets nominally worse for brain 2 with normalization, it is more constant in brain 1 [0.77-->0.76-->0.77].  We are also removing several common low expressors in "ComBat" which somewhat decreases the overall correlation.)

## Which genes are quite a bit higher in array than RNA-Seq (or vice versa)? (Both brains together)
# For this analysis we are using standard normalization, not ComBat normalization so that we can keep the genes not expressed in RNA-Seq.  The results are nearly identical using ComBat norm.

colExpr  = rep("black",length(r(datExprE)));   names(colExpr) = rownames(datExprE)
colExpr[(r(datExprE)-r(datExprRn)*1.5)>6] = "red";
rot <- prcomp( ~ r(datExprRn) + r(datExprE) )
slope <- rot$rotation[2,1] / rot$rotation[1,1]
intercept <- rot$center[2] - slope*rot$center[1]

pdf("meanExpression_betweenMethods_redProbes_F.pdf",height=10,width=9);
verboseScatterplot(r(datExprRn),r(datExprE),xlab="RSEM",ylab="Array",pch=19,col=colExpr,cex=0.5)
abline(intercept, slope,lwd=2,col="grey")  # ==> This measure is IDENTICAL with or without ComBat
abline(6,1.5,col="red")
dev.off()
```

\# (LEFT) There are several genes with high expression in microarray but not RNA-Seq, but fewer with high expression in RNA-Seq but not array. Note that we are using the highest probe as a representative probe for the gene, which means that this list represents any gene with at least one probe showing particularly high expression. We will discuss genes that have no good probes later.

**## Which genes make up this group of high array expressors? What categories do they fall in?**

write(genes[colExpr=="red"],"highArray_F.txt");

\# The high array genes consist of several groups of gene families (histones, serotonin receptors, zinc fingers, etc.), suggesting quite a bit of non-specific binding with similar genes that is not present via RNA-Seq. Note that the small number of genes high in RNA-Seq appear to be random (not shown here), suggesting that this is just noise between methods.

tmp = userListEnrichment(genes,colExpr,useBrainLists=TRUE,omitCategories="black",outputGenes=TRUE)

\# These results are fairly uninformative in this case.

**## Quantify how likely there is to find this many genes not in a gene family (based on the first three letters of the gene symbol) in a list of genes this size**

length(genes[colExpr=="red"])	# [1] 667 # Total red genes

obs = sum(table(substr(genes[colExpr=="red"],1,3))==1) # [1] 259 # Observed unique families

exp = rep(0,1000); set.seed(50)

for (i in 1:1000)	exp[i]	= sum(table(substr(genes[sample(1:length(genes),670)],1,3))==1)

2*pnorm(-abs((mean(exp)-obs)/sd(exp)))

\# [1] 8.475189e-14	# (p-value is highly significant!)

hist(exp,xlab="Expected Uniqueness", xlim=c(obs-10,max(exp))); abline(v=obs,lwd=5); abline(h=0)

\# (ABOVE RIGHT) These genes are much less unique (i.e., much more likely to be part of a gene family), than expected by chance. **It is important to note that this result is just an estimate, as my method for defining gene families is computational (based on gene symbol) and not biological.**

**## Output these genes**

\# ==> Note that we are only testing whether the most highly expressed gene for these probes are very high expressors. Other probe genes may or may not also show this pattern (more later...).

outGn = cbind(probes[colExpr=="red"],genes[colExpr=="red"])

write.csv(outGn,"VeryHighProbeExpressors.csv",row.names=FALSE)

\# Some of these I will include as examples in the supplemental figure

**## Differential expression analyses between methods (for all genes and only the high array genes)**

*# Note that the all-gene plots were generated in summaryStatsAndPlots above*

```
# There is good correlation between methods for both brains 1 (R=0.74) and 2 (R=0.78).

# Recall that the high array genes uses the standard normalization but that ComBat looks the same.
summaryStatsAndPlots(datExprE2, datExprRn, probes[colExpr=="red"], genes[colExpr=="red"], "normalizedPlots_redProbes")
```

\# (Left 2 plots) Note that these bright probes not only fail to show consistent absolute expression levels between methods, but they also fail to show consistent differential expression levels between methods. (Right 2 plots) These probes are consistent within-method, which means that, whatever they are marking, they are doing it in a consistent manner. These results suggest that these probes are not marking what they are supposed to be marking, possibly due to binding of other family members. Methods for weeding out bad probes will be discussed in more detail later.

**###################################################################################################**

**## Which method comes up with the most significant differential expression across subcortex**

*# Note that these plots were generated in summaryStatsAndPlots above*

```
# (LEFT) At better p-value thresholds, a progressively higher percentage of DEX genes are found only with RNA-Seq.  ~10% of genes are found specifically with microarray at all cutoff levels.
# (RIGHT) At all p-value cutoffs, there are more DEX genes via RNA-Seq than microarray


###################################################################################################
## What is the distribution of gene expression levels across all transcripts, on average?
# ==> This is one potential measure of "dynamic range"

datCountR2 = round(datCountR[datCountR>0.5]) # Omit "0" counts for display purposes
sum(datCountR==0)  # [1] 504375
pdf("expressionHistogram_2Methods_F.pdf",width=12,height=10); par(mfrow=c(2,1))
hist(as.matrix(datComE),xlim=c(-1,17),ylim=c(0,150000),breaks=0.25*(0:150)-8,
 main="Histogram of Array expression levels",xlab="log2(normalized intensity values)")
hist(log2(datCountR2),xlim=c(-1,17),ylim=c(0,150000),breaks=0.25*(0:150)-8,
 main="Histogram of RNA-Seq expression levels",xlab="log2(read counts)")
dev.off()
```

```
# (LEFT) There appears to be a slightly longer tail toward the low expression end for RNA-Seq data compared with microarray data (likely due to the background noise from array), which may indicate a decreased dynamic range of RNA-Seq data.  We note that we do NOT see this effect when we look at TPM values, only when we look at counts.

## Compare expression levels of genes in "replicate" samples from the same brain/region/hemisphere

samp3   = paste(s[,8],s[,9],s[,7])
pairs3  = names(table(samp3)[table(samp3)==2])
methods = c("Array","RNA-Seq")
exprCor = matrix(0,nrow=length(pairs3),ncol=length(methods));
colnames(exprCor) = methods
for (i in 1:length(pairs3)){
  samp3kp      = which(samp3==pairs3[i])
  exprCor[i,1] = cor( (datComE[,samp3kp[1]]), (datComE[,samp3kp[2]]))
  exprCor[i,2] = cor( (datComR[,samp3kp[1]]), (datComR[,samp3kp[2]]))
}
t.test(exprCor[,1],exprCor[,2])                 # t = -15.2534, df = 162.21, p-value < 2.2e-16
t.test(exprCor[,1],exprCor[,2])$ p.value        # [1] 3.728455e-33
wilcox.test(exprCor[,1],exprCor[,2])$ p.value   # [1] 1.373371e-32
# Same p-values for T-test and Wilcoxon Rank Sum tests: ~10^-32

pdf("replicateSamplePlot_F.pdf",width=3,height=5)
boxplot(exprCor,ylim=c(0.96,1), pch=19,cex=0.8)
stripchart(as.data.frame(exprCor),vertical=TRUE,add=TRUE,pch=19,cex=0.5)  # BELOW LEFT
abline(h=c(0.96,0.97,0.98,0.99,1),col="grey",lt="dotted")
dev.off()

# (ABOVE RIGHT [previous page]) Both array and RNA-seq have REALLY high replicate correlation, although RSEM is slightly (significantly) higher


###################################################################################################
## Comparison of expression reproducibility for both methods with gene size and transcript length

## Determine the correlation of each gene across mean expression levels for each subregion
# Note that this information was generated in summaryStatsAndPlots above (for ComBat)

# Brain 1, between methods:   0.438195
# Brain 2, between methods:   0.514899  # Note the better R for brain 2, which is higher quality.
# Microarray, between brains: 0.475714
# RNA-Seq, between brains:    0.627477  # Note the better R for RNA-Seq than microarray.


## Plot examples of the genes with the best between brain and between methods correlation

subregion = as.character(sampleInfo$sub_structure)
subExprE1 = findFromGroups(t(datComE[,w1]),subregion[w1])
subExprE2 = findFromGroups(t(datComE[,w2]),subregion[w2])
subExprR1 = findFromGroups(t(datComR[,w1]),subregion[w1])
subExprR2 = findFromGroups(t(datComR[,w2]),subregion[w2])
topExprE  = findFromGroups(t(datComE),toplevel) # (for later)
topExprR  = findFromGroups(t(datComR),toplevel) # (for later)
subs = colnames(subExprE1)

corrsE1R1 <- corrsE1E2 <- corrsE2R2 <- corrsR1R2 <- rep(0,dim(subExprR2)[1])
for (i in 1:dim(subExprR2)[1]) {
 corrsE1R1[i] = as.numeric(cor(as.numeric(subExprE1[i,]),as.numeric(subExprR1[i,])))
 corrsE2R2[i] = as.numeric(cor(as.numeric(subExprE2[i,]),as.numeric(subExprR2[i,])))
 corrsE1E2[i] = as.numeric(cor(as.numeric(subExprE1[i,]),as.numeric(subExprE2[i,])))
 corrsR1R2[i] = as.numeric(cor(as.numeric(subExprR1[i,]),as.numeric(subExprR2[i,])))
}
kp2 = (!is.na(corrsE1R1))&(!is.na(corrsE2R2))&(!is.na(corrsE1E2))&(!is.na(corrsR1R2))

ord = order(rowSums(-cbind(corrsE1R1,corrsE2R2,corrsE1E2,corrsR1R2)))
pdf("ExampleGenes_betweenBrainAndMethodCorrelation_F.pdf",height=10,width=9); par(mfrow=c(2,2))
for(i in c(1:50,(1:18)*1000+1)){ o = ord[i]
 m = paste(rownames(subExprR1)[o],"-",o,"-")
 verboseScatterplot(as.numeric(subExprE1[o,]),as.numeric(subExprR1[o,]),col="white",
   xlab="Array-Brain 1 (Expr.)",ylab="RNA-SEQ-Brain 1 (Expr.)",main=m)
 text(as.numeric(subExprE1[o,]),as.numeric(subExprR1[o,]),subs)
 verboseScatterplot(as.numeric(subExprE2[o,]),as.numeric(subExprR2[o,]),col="white",
   xlab="Array-Brain 2 (Expr.)",ylab="RNA-SEQ-Brain 2 (Expr.)",main=m)
 text(as.numeric(subExprE2[o,]),as.numeric(subExprR2[o,]),subs)
 verboseScatterplot(as.numeric(subExprE1[o,]),as.numeric(subExprE2[o,]),col="white",
   xlab="Array-Brain 1 (Expr.)",ylab="Array-Brain 2 (Expr.)",main=m)
 text(as.numeric(subExprE1[o,]),as.numeric(subExprE2[o,]),subs,col="blue")
 verboseScatterplot(as.numeric(subExprR1[o,]),as.numeric(subExprR2[o,]),col="white",
   xlab="RNA-SEQ-Brain 1 (Expr.)",ylab="RNA-SEQ-Brain 2 (Expr.)",main=m)
 text(as.numeric(subExprR1[o,]),as.numeric(subExprR2[o,]),subs,col="green")
}; dev.off()
```

```
# Here is one of the most significant examples, which we include in the figure as a demonstration of what the numbers mean in this metric.


## Plot these correlations in an expression level-dependent manner, using 20 quantile bins
# Note that these plots were generated in summaryStatsAndPlots above
```

```
# (LEFT) RNA-Seq wins, based on this measure (as mentioned above).  Also note that, for the lowest ~25-35% of genes, increased expression results in a consistent increase in reproducibility, at which point the relationship between gene size and reproducibility becomes much less apparent.  (RIGHT) These values correspond approximately to TPM=1 and log2(intensity)=5.


## Plot these correlations in a gene size-dependent manner, using TRANSCRIPT length

transcriptomeSizes = genesSeq[rownames(datComE),4]
genomeSizes        = genesSeq[rownames(datComE),5]
bins  = 20;   x2 = 1:bins

corrsE1E2 <- corrsR1R2 <- rep(0,dim(subExprR2)[1])
for (i in 1:dim(subExprR2)[1]) {
 corrsE1E2[i] = as.numeric(cor(as.numeric(subExprE1[i,]),as.numeric(subExprE2[i,])))
 corrsR1R2[i] = as.numeric(cor(as.numeric(subExprR1[i,]),as.numeric(subExprR2[i,])))
};  kp2 = (!is.na(corrsE1E2))&(!is.na(corrsR1R2))

sizeTest = transcriptomeSizes;     corrSizes <- meanSizes <- meanE <- meanR <- NULL;
sizes = round(as.numeric(quantile(sizeTest,(0:bins)/bins)));
for (i in x2){
 kp      = (sizeTest>=sizes[i])&(sizeTest<sizes[i+1])
 corrSizes = c(corrSizes, mean(diag(cor((datComE[kp,]),(datComR[kp,])))))
 meanSizes = c(meanSizes, median(sizeTest[kp]))
 meanE     = c(meanE, mean(r(topExprE)[kp]))
 meanR     = c(meanR, mean(r(topExprR)[kp]))
}
x = 100*x2/max(x2)

corrSizesE <- corrSizesR <- NULL
for (i in 1:bins) {
 kp = (sizeTest>=sizes[i])&(sizeTest<sizes[i+1])&kp2
 corrSizesE = c(corrSizesE, mean(corrsE1E2[kp]));
 corrSizesR = c(corrSizesR, mean(corrsR1R2[kp]))
}

pdf("transcriptSize_Vs_betweenBrainAgreement_eachMethod_F.pdf",height=6, width=15); par(mfrow=c(1,2));  ylab = "Expression correlation across brains"
xlab = "Quantile of ranked transcript size (%)"
verboseScatterplot(x,corrSizesR,main="RSEM=green, array=blue",xlab=xlab,ylab=ylab,abline=FALSE,
  type="b",ylim=c(0,0.75),col="green",pch=19);     points(x,corrSizesE,type="b",col="blue",pch=19);
abline(h= mean(corrsR1R2[kp2]),lty=2,col="green"); abline(h= mean(corrsE1E2[kp2]),lty=2,col="blue")
verboseScatterplot(x,log10(meanSizes),main=" ",xlab=xlab,ylab="log10(transcript length in bp)",
  pch=19,abline=FALSE,type="b",ylim=c(0,4))
verboseScatterplot(x,meanE,main="Array=blue, RSEM=green",xlab=xlab,pch=19,
  ylab="log2(Expression / RPKM)",abline=FALSE,type="b",col="blue",ylim=c(0,8))
points(x,meanR,type="b",col="green",pch=19);    abline(h=mean(r(topExprR)),lty=2,col="green");
abline(h=mean(r(topExprE)),lty=2,col="blue");   yl = c(0.6,0.9);
xlab = "Quantile of ranked transcript size (%)"; ylab = "Expression correlation between methods"
verboseScatterplot(x,corrSizes,main="",xlab=xlab,ylab=ylab,abline=FALSE,type="b",ylim=yl,pch=19);
dev.off()
```

\# (LEFT) As above, RNA-Seq has better between-brain agreement in expression than Microarray. For both methods, it turns out that larger genes have better between-brain agreement than shorter genes. (2^ND^) Mean gene size in each bin. (3^RD^) Genes of increasing transcript length tend to have slightly increasing expression levels, which we may not expect (especially since TPM accounts for size). (RIGHT) Within-bin correlation between methods tends to decrease slightly with increasing size. I don't know know if this means anything or is even signficant.

```
## Do larger genes tend to show more variability (as measured by max fold change) across the brain?
# ==> If so, that could explain the relationship between length and reproducibility seen by ARRAY.

varR = apply(topExprR,1,function(x) return(diff(range(x))))  # Max log2 DEX between toplevel
varE = apply(topExprE,1,function(x) return(diff(range(x))))  #  regions.
varSizesR <- varSizesE <- NULL
for (i in 1:bins) {                 kp = (sizeTest>=sizes[i])&(sizeTest<sizes[i+1])&kp2
 varSizesE = c(varSizesE, mean(varE[kp]));     varSizesR = c(varSizesR, mean(varR[kp]));
}
pdf("transcriptSize_Vs_DEXrange_eachMethod_F.pdf",height=6, width=15); par(mfrow=c(1,2))
xlab = "Quantile of ranked transcript size (%)"; ylab = "Max log2(DEX) between regions"
verboseScatterplot(x,varSizesR,main="RSEM=green, array=blue",xlab=xlab,ylab=ylab,abline=FALSE,
   type="b",ylim=c(0,2.2),col="green",pch=19);  points(x,varSizesE,type="b",col="blue",pch=19);
abline(h= mean(varR),lty=2,col="green");        abline(h= mean(varE),lty=2,col="blue")
dev.off()
```

```
# Large genes are more variable across the brain, which entirely explains the size vs. reproducibility relationship.


## Plot these correlations in a gene size-dependent manner, using GENOMIC length

sizeTest = genomeSizes;     corrSizes <- meanSizes <- meanE <- meanR <- NULL;
sizes = round(as.numeric(quantile(sizeTest,(0:bins)/bins)));
for (i in x2){
 kp      = (sizeTest>=sizes[i])&(sizeTest<sizes[i+1])
 corrSizes = c(corrSizes, mean(diag(cor((datComE[kp,]),(datComR[kp,])))))
 meanSizes = c(meanSizes, median(sizeTest[kp]))
 meanE     = c(meanE, mean(r(topExprE)[kp]))
 meanR     = c(meanR, mean(r(topExprR)[kp]))
}
x = 100*x2/max(x2)

corrSizesE <- corrSizesR <- NULL
for (i in 1:bins) {
 kp = (sizeTest>=sizes[i])&(sizeTest<sizes[i+1])&kp2
 corrSizesE = c(corrSizesE, mean(corrsE1E2[kp]));
 corrSizesR = c(corrSizesR, mean(corrsR1R2[kp]))
}

pdf("genomeSize_Vs_betweenBrainAgreement_eachMethod_F.pdf",height=6, width=15); par(mfrow=c(1,2));  ylab = "Expression correlation across brains"
verboseScatterplot(x,corrSizesR,main="RSEM=green, array=blue",xlab=xlab,ylab=ylab,abline=FALSE,
  type="b",ylim=c(0,0.75),col="green",pch=19);     points(x,corrSizesE,type="b",col="blue",pch=19);
abline(h= mean(corrsR1R2[kp2]),lty=2,col="green"); abline(h= mean(corrsE1E2[kp2]),lty=2,col="blue")
verboseScatterplot(x,log10(meanSizes),main=" ",xlab=xlab,ylab="log10(genome length in bp)",
  pch=19,abline=FALSE,type="b",ylim=c(0,6))
verboseScatterplot(x,meanE,main="Array=blue, RSEM=green",xlab=xlab,pch=19,
  ylab="log2(Expression / RPKM)",abline=FALSE,type="b",col="blue",ylim=c(0,8))
points(x,meanR,type="b",col="green",pch=19);    abline(h=mean(r(topExprR)),lty=2,col="green");
abline(h=mean(r(topExprE)),lty=2,col="blue");   yl = c(0.6,0.9);
xlab = "Quantile of ranked genome size (%)"; ylab = "Expression correlation between methods"
verboseScatterplot(x,corrSizes,main="",xlab=xlab,ylab=ylab,abline=FALSE,type="b",ylim=yl,pch=19);
dev.off()
```

```
# Pretty much the same results as with transcript length, except that the first bin is pretty bad.


## Do larger genes tend to show more variability (as measured by max fold change) across the brain?
# ==> If so, that could explain the relationship between length and reproducibility seen by ARRAY.

varR = apply(topExprR,1,function(x) return(diff(range(x))))  # Max log2 DEX between toplevel
varE = apply(topExprE,1,function(x) return(diff(range(x))))  #  regions.
varSizesR <- varSizesE <- NULL
for (i in 1:bins) {                 kp = (sizeTest>=sizes[i])&(sizeTest<sizes[i+1])&kp2
 varSizesE = c(varSizesE, mean(varE[kp]));     varSizesR = c(varSizesR, mean(varR[kp]));
}
pdf("genomeSize_Vs_DEXrange_eachMethod_F.pdf",height=6, width=15); par(mfrow=c(1,2))
xlab = "Quantile of ranked genome size (%)"; ylab = "Max log2(DEX) between regions"
verboseScatterplot(x,varSizesR,main="RSEM=green, array=blue",xlab=xlab,ylab=ylab,abline=FALSE,
   type="b",ylim=c(0,2.2),col="green",pch=19);  points(x,varSizesE,type="b",col="blue",pch=19);
abline(h= mean(varR),lty=2,col="green");        abline(h= mean(varE),lty=2,col="blue")
dev.off()
```

```
# Genome length is much more related to variability in the brain than transcriptome length, particularly with respect to very large and very small genes.  This is an interesting finding that  is tangential to focus of the manuscript and is therefore not discussed in the paper in much detail.  This would make an interesting topic of follow-up study.


###################################################################################################
## Using RNA-Seq to help identify the most biologically reliable microarray probes

## Rerun ComBat on ALL array probes (intially we only ran ComBat on the highest probe)

write.csv(dataA,"dataA_F.csv",row.names=FALSE)
pdf("ComBat_plots_allProbes_F.pdf")
datComA=ComBat("dataA_F.csv","info.txt",type="csv",write=F,par.prior=T,filter=F,skip=0,prior.plots=T)
rownames(datComA) <- probesA
dev.off();
# save(datComA, file="datComA.RData");  # Uncomment this line if you want to save this matrix
collectGarbage()
```

```
## Correlate the expression levels of each probe on the microarray (log2[intensity]) with the corresponding RNA-Seq expression levels reported using RSEM (log2[TPM+1])
# Only use the data from Brain 2, so that later Brain 1 can be treated as an independent test set.
# This will also be the basis for a probe quality control score generated later in the code.

genesCom = rownames(datComR)
useThese = is.element(genesA,genesCom);		datExprMR = datComA[useThese,]
probeMR  = probesA[useThese];			geneMR    = as.character(genesA)[useThese]
sum(useThese)/length(useThese)   # [1] 0.7487733      # ~75% of probes have matching RNA-Seq data

pCor=apply(cbind(datExprMR[,w2],datComR[geneMR,w2]),1,function(x) return(cor(x[1:116],x[117:232])))
pCor[is.na(pCor)] = 0            # To account for genes that are absent in brain 2
mean(pCor)  # [1] 0.380751       # There are pretty good correlations.
# Note that we get essentially identical results for this measure whether or not we use ComBat.


## Compare the probes with highest (best) and lowest (worst) correlation between methods to the probes identified using collapseRows (i.e., highest expression). (In brain 2)

bestProbe <- worstProbe <- highProbe <- rep("",length(genesCom))
meanExpr2  = rowMeans(datComA[useThese,w2])
for (i in 1:length(genesCom)){
 datTmp        = names(sort(pCor[is.element(geneMR,genesCom[i])]))
 worstProbe[i] = datTmp[1]
 bestProbe[i]  = datTmp[length(datTmp)]
 highProbe[i]  = names(sort(-meanExpr2[is.element(geneMR,genesCom[i])]))[1]
}
kpN = worstProbe!=bestProbe;      sum(kpN)  # [1] 17445  # Most genes have multiple probes

c(mean(pCor[worstProbe[kpN]]), mean(pCor[highProbe[kpN]]), mean(pCor[bestProbe[kpN]]))
# [1] 0.2503650 0.4212420 0.4883123
# We can definitely improve the between-method agreement by appropriately choosing probes

sum(bestProbe[kpN]==highProbe[kpN])/sum(kpN)     # [1] 0.6013
sum(worstProbe[kpN]==highProbe[kpN])/sum(kpN)    # [1] 0.3073
# A majority of the best probes are also the ones with highest expression


## Example genes with *different* worse, highest, and best probes, where at least one probe is of good quality and at least one is of bad quality.

numDiffProbe = apply(cbind(bestProbe,highProbe,worstProbe),1,function(x) return(length(unique(x))))
passFailProbe= (pCor[worstProbe]<0.1)&(pCor[bestProbe]>0.8)&(numDiffProbe==3)

pdf("exampleProbesForFig4.pdf",height=4,width=11)
for (i in which(passFailProbe)){ par(mfrow=c(1,3));
verboseScatterplot(datComR[i,w2],datExprMR[worstProbe[i],w2],main=worstProbe[i],pch=19,
  xlab="RNA-Seq",ylab="Array",col="#444444");
verboseScatterplot(datComR[i,w2],datExprMR[highProbe[i],w2],main=highProbe[i],pch=19,
  xlab=paste("RNA-Seq –",rownames(datComR)[i]),ylab="Array",col="#888888");
verboseScatterplot(datComR[i,w2],datExprMR[bestProbe[i],w2],main=bestProbe[i],pch=19,
  xlab="RNA-Seq",ylab="Array",col="#CCCCCC");
}; dev.off()
```

```
# ZFR2 (page 54) is a good example of the extreme case where the three probes would give you three different results (negative, no, and positive correlation with RNA-Seq, respectively).

## For each gene, plot the best agaist the worst probe correlation

plot(sort(pCor[bestProbe[kpN]]-pCor[worstProbe[kpN]]),xlab="",ylab="R range",type="l",lwd=3,ylim=c(0,1))
abline(h=c(0.1,0.2,0.3,0.4), lty="dotted")  # (LEFT)
hist(sort(pCor[bestProbe[kpN]]-pCor[worstProbe[kpN]]),xlab="R range")  #(RIGHT)
```

```
# Choice of probe can make a big difference (for 43% of genes, [R range]>0.2, for 29% it is >0.3)


## Peform all comparisons for these three sets of probes.

summaryStatsAndPlots(datComA[worstProbe,],datComR, worstProbe,genesCom, "worstProbe_plots")
collectGarbage()
summaryStatsAndPlots(datComA[highProbe,], datComR, highProbe, genesCom, "highProbe_plots")
collectGarbage()
summaryStatsAndPlots(datComA[bestProbe,], datComR, bestProbe, genesCom, "bestProbe_plots")
collectGarbage()


## Average expression levels calculated by microarray vs. RNA-Seq
#          WORST PROBE                   HIGHEST PROBE                    BEST PROBE
```

```
# Improvements in reproducibility do not necessarily imply improved agreement in absolute expression levels between methods.


## Differential expression between METHODS in brain 2: correlation of log2(expression calls)
#          WORST PROBE                   HIGHEST PROBE                    BEST PROBE
```

```
## Differential expression between METHODS in brain 1 (our independent "test" brain)
#          WORST PROBE                   HIGHEST PROBE                    BEST PROBE
```

\# Unsurprisingly, when we define "best probe" based on highest correlation across samples between methods, we also find more consistent fold changes between regions in brain 2 between methods, since these are related tests. Potentially of more interest is that we also find better between-method agreement in the test brain.

**## Differential expression between brains in microarray: correlation of log2(FC) between regions**

\#	WORST PROBE	HIGHEST PROBE	BEST PROBE

```
# Probes that show better correlation between methods, also show better agreement between brains, suggesting that it is reasonable to use agreement with RNA-Seq as a measurement for probe quality.  But note that the highest probes do nearly as well by this measure, even though only ~60% agree.

###################################################################################################
## Can we use the RNA-Seq information to filter out unreliable probes and make a more accurate
##   estimate of absolute gene expression levels with microarrays?

# ==> Note that for this analysis, we are using TbT normalized data, NOT ComBat normalized data, as that might affect the absolute expression levels output by RNA-Seq.  Also recall the the analysis above produces the SAME results when using pre- or post-ComBat normalized data.

## Get estimated linear fit (using the 5% and 95% quantiles) and correlations
# ==> Note that this function also returns p and q values for correlation significance.  This is what we will use to determine whether a probe passes.

probeMR    = as.character(probeMR)
datExprEmr = dataA[probeMR,];        datComEmr = datComA[probeMR,] # ComBat data
datExprRmr = datExprRn[geneMR,];     datComRmr = datComR[geneMR,]  # ComBat data
(globScale =  mean(as.matrix(dataA)[,w2]))  # [1] 5.249917
datExprEmr = datExprEmr-globScale;   datComEmr = datComEmr-globScale;
# Note: The global scaling step accounts for general "brightness" of the arrays, which varies from experiment to experiment and is not accounted for in the linear fit.  This step is important.

datRA2     = cbind(datExprRmr[,w2],datExprEmr[,w2])                # Note the pre-ComBAT data
varLabel   = list(1:116,117:232)
getEstFitforApply <- function(x) {  xR = x[varLabel[[1]]];  xA = x[varLabel[[2]]]
 qR = as.numeric(quantile(xR,c(0.05,0.95)));  qA = as.numeric(quantile(xA,c(0.05,0.95)));
 if(abs(qR[1]-qR[2])<0.00001) return(c(1,0,0,1,qA,qR))
 m = (qA[2]-qA[1])/(qR[2]-qR[1]);    b = qA[1]-m*qR[1];    cr = cor.test(xR,xA)
 return(c(m,b,as.numeric(cr$est),as.numeric(cr$p.val),qA,qR))
}
datLE = t(apply(as.matrix(datRA2),1,getEstFitforApply))
colnames(datLE) = c("m","b","r","pval","a05","a95","r05","r95")
rownames(datLE) = probeMR
qvals = qvalue(datLE[,4])$qvalues
passGenes = (qvals<0.1)&(datLE[,3]>0)  # Keep genes with qvalue < .1
datLE2 = datLE;  datLE = datLE[passGenes,]
# We note that genes that fail could fail either because they are of low quality, or because they show very little biological variability across the brain.  In this case, the there would not be enough signal to properly correlate between methods.

bestProbeP = intersect(bestProbe,rownames(datLE))
bestGeneP  = genesA[bestProbeP]
length(bestProbeP) / length(bestProbe)
# [1] 0.8179216  # we lose ~18% of possible genes this way.


## Repeat all of the above comparisons using only the best probes that passed the filtering step.
# ==> Also the ones that failed, for comparison

bestGeneP = as.character(genesA[bestProbeP])
summaryStatsAndPlots(datComA[bestProbeP,],datComR[bestGeneP,],bestProbeP, bestGeneP,
           "passingBestProbe_plots");
collectGarbage()
bestProbeF = setdiff(bestProbe,bestProbeP)
bestGeneF  = as.character(genesA[bestProbeF])
summaryStatsAndPlots(datComA[bestProbeF,],datComR[bestGeneF,],bestProbeF, bestGeneF,
           "failingBestProbe_plots");
collectGarbage()


## Plot some of the correlations in the format that will be used in the paper
# ==> The numbers listed below were taken from the "stats.txt" file in the relevent folders.

pdf("comparisonBarPassVsFail.pdf",height=7,width=5)
ganP = cbind(c(0.6439, 0.7901, 0.8470, 0.8725, 0.0419), c(0.7618, 0.8525, 0.8600, 0.8790, 0.4527))
colnames(ganP) = c("DEX Cor (method, B2)", "DEX Cor (brain)")
rownames(ganP) = c("worst","highest","best","passing","failing")
barplot(ganP,beside=TRUE,ylim=c(0,1),ylab="Correlation",col=c("#444444","#888888",
   "#CCCCCC","green","red"));  abline(h=0.9,lty="dashed",col="grey");
dev.off()
```

```
# (LEFT) Both of the differential expression measures improve when we remove disagreeing probes.

## Plot the expression level histograms
pdf("comparisonExpressionHistogramPassVsFail.pdf",height=7,width=7)
meanMR   = rowMeans(datExprMR[,w2])
histPass = hist(meanMR[bestProbeP],plot=FALSE,breaks=0:17)$counts
histFail = hist(meanMR[bestProbeF],plot=FALSE,breaks=0:17)$counts
counts   = rbind(histPass,histFail)
nm = paste(0:16,1:17,sep="-")
barplot(counts,main="Probes passing vs. expression level", xlab="Expression levels",names.arg=nm,
  ylab="Count",col=c("green","red"),legend =c("Passed QC","Failed QC"),ylim=c(0,2500),las=2)
dev.off()
# (ABOVE RIGHT) Most of the failing probes have very low expression, suggesting they may represent genes with low expression in the brain, rather than probes which to not work.

length(bestProbeP[meanMR[bestProbeP]>3]) / length(bestProbe[meanMR[bestProbe]>3])   # [1] 0.9116378
length(bestProbeP[meanMR[bestProbeP]<3]) / length(bestProbe[meanMR[bestProbe]<3])   # [1] 0.4684547
c(length(bestProbeF),length(bestProbeP)) # [1]  3371 15143
# If we only consider probes expressed with log2(intensity)>3, then we have fewer failing probes, but over half fail with low expression


## How do these parameters look?

par(mfrow=c(1,6))
rangeA = datLE[,6]-datLE[,5];   rangeR = datLE[,8]-datLE[,7]
hist(rangeA,xlim=c(0,4),main="Array range",xlab="Qu95%-Qu5%",ylab="Count")
hist(rangeR,xlim=c(0,4),main="RSEM range",xlab="Qu95%-Qu5%",ylab="Count")
hist(log2(datLE[,1]),xlim=c(-1,5),main="Multiplier",xlab="log2(m)",ylab="Count")
hist(datLE[,2],xlim=c(-15,5),main="Offset",xlab="b",ylab="Count")
hist(datLE[,3],xlim=c(0,1),main="Correlation",xlab="r",ylab="Count")
hist(-log10(datLE[,4]+0.0000000000000000001), main="P-value",xlab="-log10(p)",ylab="Count")
```

```
## Output parameters (and other info) to file for publication as Supplementary Table 1

datLE3 = matrix(NA,nrow=length(probesA), ncol=12)
colnames(datLE3) = c(colnames(datLE2),"Gene","MeanExprArray","qVals","Pass")
rownames(datLE3) = probesA
datLE3[rownames(datLE2),1:8] = datLE2
datLE3[,9] = genesA
datLE3[,10] = rowMeans(dataA)
datLE3[names(qvals),11] = qvals
datLE3[names(qvals),12] = passGenes
write.csv(datLE3,"SupplementaryTable1_parametersForAgilentArray.csv",row.names=TRUE)

## Scale the pre-ComBat data, then rerun ComBat on the scaled array data.
# ==> This is done to retain independence between brains

datExprEmr_LE = t(apply(cbind(datLE2[,1:2],datExprEmr),1,function(x) return((x[3:233]-x[2])/x[1])))
write.csv(datExprEmr_LE,"dataEmr_LE_F.csv",row.names=FALSE)  # Uncomment this line
datComEmr_LE = ComBat("dataEmr_LE_F.csv","info.txt",type="csv",write=F,par.prior=T,filter=F,skip=0,
  prior.plots=T);
rownames(datComEmr_LE) <- rownames(datExprEmr_LE)
save(datComEmr_LE,file="ComBat_LEscaled_data.RData") # Uncomment this line to save file


## Plot an example gene and before and after correction and calculate some stats for the figure
# ==> Use SATB2 (A_32_P174365), although this could be changed.

## For plots, undo global scaling.  Note that this does NOT affect the results after scaling.
datLEgs = datLE2;   datLEgs[,c(2,5,6)] = datLE2[,c(2,5,6)]+globScale

kpP = probeMR=="A_32_P174365"
pdf("SATB2_examplePlot.pdf",height=6,width=5.5)
verboseScatterplot(datComRmr[kpP,],datComEmr[kpP,]+globScale,xlim=c(0.5,8.5),ylim=c(0.5,8.5),
  main="SATB2",xlab="RNA-Seq",ylab="Array",pch=19,col="grey")
points(datLEgs[kpP,7:8],datLEgs[kpP,5:6],cex=3,lwd=2,col="red",pch=19)
abline(datLEgs[kpP,2],datLE2[kpP,1],col="grey");
points(datComRmr[kpP,],datComEmr_LE[kpP,],pch=19)
points(datLEgs[kpP,7:8], datLEgs[kpP,7:8],cex=3,lwd=2,col="red",pch=19); abline(0,1);
dev.off()
```

```
m=quantile(datLE[,1],c(0.05,0.25,0.5,0.75,0.95))#        5%       25%       50%       75%       95%
                                                # 0.7718922 1.0283897 1.3066877 1.7724292 3.2802797
b=quantile(datLE[,2],c(0.05,0.25,0.5,0.75,0.95))+globScale   # (Add global scaling)
                                                #        5%       25%       50%       75%       95%
                                                # -6.302978 -0.964555  1.088914  2.719434  5.146623

pdf("scaleBarMB.pdf",height=4,width=2.3)
errbar(c(0,1),y=c(m[3],b[3]),yplus=c(m[5],b[5]),yminus=c(m[1],b[1]),pch="-", cap=0.25,
  xlab="",ylab="",main="Parameter range",xlim=c(-0.5,1.5),ylim=c(-1.2,3.2))
errbar(c(0,1),y=c(m[3]-0.1,b[3]-0.1),yplus=c(m[4],b[4]),yminus=c(m[2],b[2]),pch="-",cex=4,
  lwd=3,cap=0.25,add=TRUE);  # (I need the -0.1 because the errbar/pdf functions mess up the plot)
abline(h=c(0,1),col="grey");
dev.off()
# (Plot is above right.)


## Repeat all of the above comparisons on the SCALED data, using only the passing probes.

datBestE    = datComA[bestProbeP,];            datBestEb    = dataA[bestProbeP,]
datBestR    = datComR[bestGeneP,];             datBestRb    = datExprRn[bestGeneP,]
datBestE_LE = datComEmr_LE[bestProbeP,];       datBestE_LEb = datExprEmr_LE[bestProbeP,];

summaryStatsAndPlots(datBestE_LE,datBestR,bestProbeP,bestGeneP,"scaledProbe_plots")
collectGarbage()

## How do these scaled results compare to the unscaled results

# ==> The numbers listed above were taken from the "stats.txt" file in the relevent folders.
pdf("scaledVsUnscaledComparisonBarPlots.pdf",height=7,width=4)
ganA2 = cbind(c(0.8725, 0.89313), c(0.8790, 0.89925))
colnames(ganA2) = c("DEX Cor (method)","DEX Cor (brain)"); rownames(ganA2) = c("Unscaled","Scaled")
barplot(ganA2,beside=TRUE,ylim=c(0,1),ylab="Correlation",col=c("green","black"));
abline(h=0.9,lty="dashed",col="grey");  dev.off()
# (BELOW LEFT) Note that both parameters are improved with this scaling.


## What are the parameters for the subset of "best" probes used in this analysis?

m=quantile(datLE[bestProbeP,1],c(0.05,0.25,0.5,0.75,0.95))
#        5%       25%       50%       75%       95%
# 0.7979971 1.0381239 1.3080376 1.7844625 3.6936756
b=quantile(datLE[bestProbeP,2],c(0.05,0.25,0.5,0.75,0.95))+globScale
#        5%       25%       50%       75%       95%
# -4.646362  0.035780  1.577566  3.285742  5.502733
# Overall, the parameters are basically the same, except that there are slightly fewer genes with negative intercept after removing global scaling (which makes sense--> you can't have negative expression).

## Replot the mean absolute expression correlations between array and RNA-Seq
# Note that this information was generated in summaryStatsAndPlots above (for ComBat)
```

```
# (RIGHT) Scaling improves between between-method absolute expression calls in both brain 2 (as expected based on the methodology), as well as brain 1 (which was not guaranteed, as none of these data were used to determine parameters, although might be imposed by running ComBat).


## What are the correlations in each individual sample if we don't use ComBat data?

rArr <- rLE <- NULL
for (i in 1:length(samps)){
 rArr = c(rArr,cor(datBestRb[,i],datBestEb[,i]))
 rLE  = c(rLE,cor(datBestRb[,i],datBestE_LEb[,i]))
}
rAll = c(rArr,rLE)
rNM  = paste(c(rep("Norm",231),rep("Qscal",231)),rep(whichBrain,2),sep="")
verboseBoxplot(rAll,rNM,las=2,main="Between method corr",ylab="R",xlab="",notch=FALSE)
```

```
# These results hold in EVERY sample from both brains, which is NOT an artifact of the methodology (Recall that the parameters were selected only using samples from Brain 2, and NOT brain 1).
## Now plot all of these samples FROM BRAIN 1 (the test brain) individually

pdf("plotAllBrain1SSMIconversions.pdf",height=6,width=6)
plot(rArr[w1][order(rArr[w1])],pch=19,col="grey",ylim=c(0.6,1),ylab="Between-method correlation")
abline(h=0.95,lty="dashed",col="grey",lwd=2);  points(rLE[w1][order(rArr[w1])],pch=19);
dev.off()
```

```
# (LEFT) Correlations are much better for all samples.

## Show a before and after example.  Use the arrow above (index 12) as an example.

pdf("beforeToAfterConversionPlotsBrain1.pdf",height=6,width=10.6)
i=12;   xy = c(-2,16);     par(mfrow=c(1,2))
verboseScatterplot(datBestRb[,i],datBestEb[,i],xlab="RSEM (TPM)",ylab="Array (intensity)",
 xlim=xy,ylim=xy,pch=19,cex=0.3,col="darkgrey");
abline(h=0);abline(v=0);abline(0,1,lty="dashed",col="black",lwd=2)
verboseScatterplot(datBestRb[,i],datBestE_LEb[,i],xlab="RSEM (TPM)",ylab="Array (mTPM)",
 xlim=xy,ylim=xy,pch=19,cex=0.3); abline(h=0);abline(v=0);abline(0,1,lty="dashed",col="grey",lwd=2)
dev.off()
# (ABOVE RIGHT).  These look good!  Note the better correlation, thinner black band of dots, and the fact that these dots are hugging the X=Y line (indicating agreement with RNA-Seq values).

# But what if we look at data generated in different tissue on microarrays processed in a different facility?


################################################################################################
## Determine how well these parameters apply to human ESCs from H1 and H9 cell lines

## Take the subset of these data corresponding to the best probes above.
# ==> Note that these data are already in log2 space

(globScaleESC  = mean(as.matrix(datESCA)))
# [1] 6.428423     # Higher average intensity than in brain
# Omit missing genes and genes with no expression in RNA-Seq
kpESC    = is.element(bestGeneP,rownames(datESCR[rowSums(datESCR)>0,]))
geneESC  = bestGeneP[kpESC]
probeESC = bestProbeP[kpESC]
datESCR  = datESCR[geneESC,]
datESCA  = datESCA[probeESC,]

## Load conversion file that was generated above based on brain 2, and convert ESC array data

arraytorsem  = read.csv("SupplementaryTable1_parametersForAgilentArray.csv",as.is=T,row.names=1);
arraytorsem  = arraytorsem[probeESC,]
datESCAScale = datESCA-globScaleESC;
datESCAScale = (datESCAScale-arraytorsem$b)/arraytorsem$m;
sampESC      = colnames(datESCR)

## Find the and plot correlations between method before and after scaling

compCorU = rep(0, length(sampESC));    names(compCorU) = sampESC;    compCorS = compCorU
for (i in 1:length(compCorU)){
 compCorU[i] = cor(datESCA[,i],datESCR[,i]);
 compCorS[i] = cor(datESCAScale[,i],datESCR[,i]);
}
cbind(compCorU,compCorS) #              compCorU  compCorS
                         # YW.H1.d0.1a 0.7926831 0.9023110
                         # ST.H9.d0.2a 0.8181572 0.9090834

pdf("meanExpression_betweenMethods_humanESC_day0_F.pdf",height=11,width=10);
xy = c(-2,17);     par(mfrow=c(2,2))
verboseScatterplot(datESCR[,1],datESCA[,1],main="H1 (day 0, unscaled)",pch=19,cex=0.5,
  ylab="Microarray (intensity)",xlab="RNA-Seq (TPM)",xlim=xy,ylim=xy,col="darkgrey");
  abline(h=0);abline(v=0);abline(0,1,lty="dashed",col="black",lwd=2)
verboseScatterplot(datESCR[,1],datESCAScale[,1],main="H1 (day 0, scaled)",pch=19,cex=0.5,
  ylab="Microarray (mTPM)",xlab="RNA-Seq (TPM)",xlim=xy,ylim=xy,col="black");
  abline(h=0);abline(v=0);abline(0,1,lty="dashed",col="grey",lwd=2)
verboseScatterplot(datESCR[,2],datESCA[,2],main="H9 (day 0, unscaled)", pch=19,cex=0.5,
  ylab="Microarray (intensity)",xlab="RNA-Seq (TPM)",xlim=xy,ylim=xy,col="darkgrey");
  abline(h=0);abline(v=0);abline(0,1,lty="dashed",col="black",lwd=2)
verboseScatterplot(datESCR[,2],datESCAScale[,2],main="H9 (day 0, scaled)",pch=19,cex=0.5,
  ylab="Microarray (mTPM)",xlab="RNA-Seq (TPM)",xlim=xy,ylim=xy,col="black");
  abline(h=0);abline(v=0);abline(0,1,lty="dashed",col="grey",lwd=2)
dev.off()
```

```
# These data the scaling factors do a very good job of improving the between-method agreement, and a pretty good job of aligning the absolute expression levels, although not as good as with the brain 1 and brain 2 data.  This is not surprising, given the differences between methodologies.


################################################################################################
## Which method comes up with the most significant differential expression across NEOCORTEX

varLabels = as.character(sampleInfo$main_structure)[cortexType]
anovaPEp  = apply(datBestE[,cortexType],1,getAnovaPvalforApply)
anovaPQp  = apply(datBestE_LE[,cortexType],1,getAnovaPvalforApply)
anovaPRp  = apply(datBestR[,cortexType],1,getAnovaPvalforApply)

pThresh = c(1:5,(3:6)*2);     aOnly <- rOnly <- both <- pThresh*0;
for (i in 1:length(pThresh)){
  p       = 10^(-pThresh[i]);   isR      = anovaPRp<p;         isA      = anovaPQp<p
  both[i] = sum(isA&isR);       aOnly[i] = sum(isA&(!isR));    rOnly[i] = sum(isR&(!isA))
}
total = both+aOnly+rOnly;      datPlot = 100*rbind(both/total,rOnly/total,aOnly/total)
colnames(datPlot) = pThresh;   rownames(datPlot) = c("Common","RNA-Seq","Array")
barplot(datPlot,beside=FALSE,ylab="% of DEX genes",xlab="-log10(p-value)",ylim=c(0,100),
 col=c("#000000","#888888","#DDDDDD"))
```

```
# (LEFT) In cortex, there is much less agreement between DEX genes and more DEX genes in RNA-Seq at nearly every p-value cutoff.

# How many total DEX genes are found with each method?
datPlot = rbind(rOnly+both,aOnly+both)
colnames(datPlot) = pThresh;   rownames(datPlot) = c("RNA-Seq","Array")
barplot(datPlot,beside=TRUE,ylab="# genes DEX",xlab="-log10(p-value)",
 col=c("#888888","#DDDDDD"));
for (i in 1:length(both)){ y = both[i]; x = i*3-2.3; segments(x,y,x+2.6,y,lwd=3) }
# (ABOVE RIGHT) # At all p-value cutoffs, there are more DEX genes via RNA-Seq than microarray.  Also note that there tend to be fewer genes DEX in cortex than subcortex (compare with other doc).

## What is the overlap (for Venn Diagram) using a strict Bonferroni correction of p=0.05?

(p = 0.05 / length(anovaPEp))   # [1] 3.301856e-06
isR = anovaPRp<p;   isQ  = anovaPQp<p;  isE  = anovaPEp<p
library(gplots)
venn(as.data.frame(cbind(isR,isE,isQ)))
```

```
# Note the additional 120 genes overlapping with RNA-Seq that we pick up after scaling.
###################################################################################################
## What is the minumum number of samples for which we can relaibly use the RNA-Seq information to
## filter out unreliable probes and estimate of absolute gene expression levels with microarrays?

## Read in the original parameters that were generated using all of the data

paramOrig = read.csv("SupplementaryTable1_parametersForAgilentArray.csv",row.names=1)
paramOrig = paramOrig[probeMR,]   # Only consider probes with corresponding RNA-Seq genes

## Pick a sample subset using the following strategy:
# 1) Iteratively remove the most similar sample and regenerate parameters to roughly order samples
# 2) Using this order as base-line, choose replicates by alternating between brain regions

#    We use these plots as reference for determining the order of the toplevel regions:
```

```
# Note that we are not claiming this is the best method for choosing samples, but it seems to work for us.  If one were to optimize sample selection, the conclusions from this analysis would either stay the same or get better, but would NOT get worse.  Therefore, our results could be considered a lower bound of results.

kpOrder = NULL
datUse  = datExprRmr[,w2]
for (i in 1:114) {
 kp      = !is.element(colnames(datUse),kpOrder)
 corTmp  = cor(datUse[,kp],datUse[,kp])
 diag(corTmp) = 0
 mnCor   = apply(corTmp,1,mean)
 top2    = c(which.max(corTmp)%%dim(corTmp)[1],ceil(which.max(corTmp)/dim(corTmp)[1]))
 top1    = names(mnCor[top2][order(-mnCor[top2])])[1]
 kpOrder = c(kpOrder,top1)
}
kpOrder = c(kpOrder,setdiff(colnames(datUse),kpOrder))
kpOrder = kpOrder[116:1]

(tops = unique(toplevel)[c(10,9,6,7,8,4,3,2,5,1)])
#[1] "CbCx" "GP"   "PHG"  "Str"  "CgG"  "Ins"  "OL"   "FL"   "TL"   "PL"
toplevel2 = toplevel;              names(toplevel2) = colnames(datExprR)
kpOrder2 = NULL
for (i in 1:3) for (t in tops) kpOrder2 = c(kpOrder2,toplevel2[kpOrder][toplevel2[kpOrder]==t][i])
kpOrder2 = names(kpOrder2)
kpOrder2 = c(kpOrder2,setdiff(kpOrder,kpOrder2)) # Put the rest of the samples back in the analysis


## Determine the parameters using the top 3-10, 12, 14, 16, 18, 20, 22, 25, 30, and ALL samples
# For plotting purposes, we show the "ALL" samples data at the location of N=35

# Summarize some of the steps from the analysis above into a single function for simplicity
getEstFitforApply <- function(x) {  xR = x[varLabel[[1]]];  xA = x[varLabel[[2]]]
 qR = as.numeric(quantile(xR,c(0.05,0.95)));  qA = as.numeric(quantile(xA,c(0.05,0.95)));
 if(abs(qR[1]-qR[2])<0.00001) return(c(1,0,0,1,qA,qR))
 m = (qA[2]-qA[1])/(qR[2]-qR[1]);    b = qA[1]-m*qR[1];    cr = cor.test(xR,xA)
 return(c(m,b,as.numeric(cr$est),as.numeric(cr$p.val),qA,qR))
}

paramsNewByDistance = paramsNew
count     = c(3:10,12,14,16,18,20,22,25,30)
paramsNew = list()
datRA2am  = as.matrix(datRA2)

for (ct in 1:length(count)){
 kpIndex  = is.element(colnames(datUse),kpOrder2[1:count[ct]])
 varLabel = list((1:116)[kpIndex],(117:232)[kpIndex])
 datLE    = t(apply(datRA2am,1,getEstFitforApply))
 colnames(datLE) = c("m","b","r","pval","a05","a95","r05","r95")
 rownames(datLE) = probeMR
 paramsNew[[ct]] = datLE
 write(paste("Done with",count[ct]),"")
}
names(paramsNew) = as.character(count)


## Compare parameters and resulting between-method stats with those found using all the data

## How much of a drop-off in overall range (q95 – q05) in array with decreasing number of samples?
count2 = c(count,35)
meanRange <- sdRange <- rep(0,length(count2))
meanRange[length(count2)] = mean(paramOrig[,6]-paramOrig[,5])
sdRange[length(count2)]   = sd(paramOrig[,6]-paramOrig[,5])
for (ct in 1:length(count)){
  meanRange[ct] = mean(paramsNew[[ct]][,6]-paramsNew[[ct]][,5])
  sdRange[ct]   = sd(paramsNew[[ct]][,6]-paramsNew[[ct]][,5])
}

## How much of a drop-off in q-values with decreasing number of samples?
for (ct in 1:length(count)){
  paramsNew[[ct]][is.na(paramsNew[[ct]][,4]==TRUE),4] = 1
  paramsNew[[ct]] = cbind(paramsNew[[ct]],qvalue(paramsNew[[ct]][,4])$qvalues)
}
totQ  = meanRange*0;   qvals = qvalue(paramOrig[,4])$qvalues
totQ[length(count2)] = sum((qvals<0.1)&(datLE[,3]>0))
for (ct in 1:length(count))
  totQ[ct] = sum((paramsNew[[ct]][,9]<0.1)&(paramsNew[[ct]][,3]>0))

## Repeat, but this time showing the percent of total genes identified.
fracQ = totQ*0;
qvals = qvalue(paramOrig[,4])$qvalues;  kp = (qvals<0.1)&(paramOrig[,3]>0)
fracQ[length(count2)] = length(intersect(bestProbe,rownames(paramOrig)[kp]))/length(bestProbe)
for (ct in 1:length(count)){
 qvals = qvalue(paramsNew[[ct]][,4])$qvalues;  kp = (qvals<0.1)&(paramsNew[[ct]][,3]>0)
 fracQ[ct] = length(intersect(bestProbe,rownames(paramsNew[[ct]])[kp]))/length(bestProbe)
}

## How much of a drop-off in mean correlation with decreasing number of samples?
meanCorr <- sdCorr <- rep(0,length(count2))
meanCorr[length(count2)] = mean(paramOrig[,3])
sdCorr[length(count2)]   = sd(paramOrig[,3])
for (ct in 1:length(count)){
  meanCorr[ct] = mean(paramsNew[[ct]][,3])
  sdCorr[ct]   = sd(paramsNew[[ct]][,3])
}

## Plot everything below!
pdf("findingMinimumSamplesToRunPlots.pdf",height=5,width=20)
par(mfrow=c(1,4))
plot(count2,meanRange, ylab="Mean Range",xlab="# Samples",pch=19,ylim=c(0,3),cex=2)
points(count2,meanRange+sdRange,pch=19); points(count2,meanRange-sdRange,pch=19)
# (LEFT) Using these hand-picked samples, we can do as well with 5-10 samples, as we can with all.

plot(count2,totQ, ylab="# passing probes",xlab="# Samples",pch=19,cex=2)
totQ[11]/totQ[17]  # [1] 0.8077232  # With 16 samples we get 81% of the probes with 116
# (LEFT CENTER) With this parameter, we still need 15-20 to get enough statistical power for a lot of the genes to pass the q-value threshold, even if the differential expression is high enough.

plot(count2,100*fracQ, ylab="% probes with passing gene",xlab="# Samples",pch=19,cex=2)
fracQ[11]/fracQ[17]  # [1] 0.8351053  # With 16 samples we get ~84% of the genes with 116
fracQ[11]            # [1] 0.6830507
# (RIGHT CENTER) With this parameter, we still need 15-20 to get enough statistical power for a lot of the genes to pass the q-value threshold, even if the differential expression is high enough.

plot(count2,meanCorr, ylab="Mean Correlation",xlab="# Samples",pch=19,ylim=c(-0.2,1),cex=2)
points(count2,meanCorr+sdCorr,pch=19); points(count2,meanCorr-sdCorr,pch=19)
# (RIGHT) Based on correlation, even with only 3 samples, we are already as good as with all samples, on average.
dev.off()
```

```
## In conclusion, with our data, although it is clearly better to use more samples, by carefully choosing ~15-20 samples we can perform our probe QC reasonably close to as well as by using all 116 samples.  We hypothesize that with more diverse set of samples, we could do significantly better.

## Try reproducing some of the biological results from the paper using parameters generated with the hand-picked, smaller number of samples

paramsNew[["116"]] = as.matrix(paramOrig[,c(1:8,11)])
count = c(5,16,116)         # Note that this line could easily be adapted to include ALL of the subsets above, but we are only showing two subsets since that is all we discuss in the paper.

###################### START OF LOOP ######################
for (ct in 1:length(count)){

## Get the right subset of the data
folder   = paste("subset",count[ct],"samples",sep="_")
datLEtmp = paramsNew[[as.character(count[ct])]]
scale2   = -datLEtmp[,3]

## Find the best probe for each gene based on correlation
bestProbe <- rep("",length(genesCom))
for (i in 1:length(genesCom)){
 datTmp       = names(sort(scale2[is.element(geneMR,genesCom[i])]))
 bestProbe[i] = datTmp[1]
}
datTmpScale = t(apply(cbind(datLEtmp[,1:2],datExprEmr),1,function(x) return((x[3:233]-x[2])/x[1])))
datTmpScale = datTmpScale[bestProbe,]

## ComBat-normalize the data
write.csv(datTmpScale,"dataTmp.csv",row.names=FALSE)  # Uncomment this line
datTmpScaleCom = ComBat("dataTmp.csv","info.txt",type="csv",write=F,par.prior=T,filter=F,skip=0,
  prior.plots=T);
rownames(datTmpScaleCom) <- rownames(datTmpScale)

## Define some useful data variables
bestGene   = genesA[bestProbe]
datComEtmp = datComA[bestProbe,];            datNormEtmp = dataA[bestProbe,]
datComRtmp = datComR[bestGene,];             datNormRtmp = datExprRn[bestGene,]

## Calculate all the standard plots the scaled array data
summaryStatsAndPlots(datTmpScaleCom,datComRtmp,bestProbe,bestGene,folder)
collectGarbage()

## Find the correlations in each individual sample if we don't use ComBat data?
rArr <- rLE <- NULL
for (i in 1:length(samps)){    rArr = c(rArr,cor(datNormRtmp[,i],datNormEtmp[,i]))
                               rLE  = c(rLE,cor(datNormRtmp[,i],datTmpScale[,i]))  }
rAll = c(rArr,rLE)
rNM  = paste(c(rep("Norm",231),rep("Qscal",231)),rep(whichBrain,2),sep="")
pdf(paste(folder,"/absExprSummary.pdf",sep=""), height=5, width=7)
verboseBoxplot(rAll,rNM,las=2,main="Between method corr",ylab="R",xlab="",notch=FALSE)
dev.off()

## Now plot all of these samples FROM BRAIN 1 (the test brain) individually
pdf(paste(folder,"/plotAllBrain1SSMIconversions.pdf",sep=""),height=6,width=6)
plot(rArr[w1][order(rArr[w1])],pch=19,col="grey",ylim=c(0.6,1),ylab="Between-method correlation")
abline(h=0.95,lty="dashed",col="grey",lwd=2);  points(rLE[w1][order(rArr[w1])],pch=19);
dev.off()

## Show a before and after example (index 12, for consistency with paper)
pdf(paste(folder,"/beforeToAfterConversionPlotsBrain1.pdf",sep=""),height=6,width=10.6)
i=12;   xy = c(-2,16);     par(mfrow=c(1,2))
verboseScatterplot(datNormRtmp[,i],datNormEtmp[,i],xlab="RSEM (TPM)",ylab="Array (intensity)",
 xlim=xy,ylim=xy,pch=19,cex=0.3,col="darkgrey");
abline(h=0);abline(v=0);abline(0,1,lty="dashed",col="black",lwd=2)
verboseScatterplot(datNormRtmp[,i],datTmpScale[,i],xlab="RSEM (TPM)",ylab="Array (mTPM)",
 xlim=xy,ylim=xy,pch=19,cex=0.3); abline(h=0);abline(v=0);abline(0,1,lty="dashed",col="grey",lwd=2)
dev.off()

## Now repeat some of the above comparisons, only including passing probes (using VERY weak criteria for passing)

pass = (datLEtmp[bestProbe,4]<0.8)&(datLEtmp[bestProbe,3]>0.1)

## Find the correlations in each individual sample if we don't use ComBat data?
rArr <- rLE <- NULL
for (i in 1:length(samps)){    rArr = c(rArr,cor(datNormRtmp[pass,i],datNormEtmp[pass,i]))
                               rLE  = c(rLE,cor(datNormRtmp[pass,i],datTmpScale[pass,i]))  }
rAll = c(rArr,rLE)
rNM  = paste(c(rep("Norm",231),rep("Qscal",231)),rep(whichBrain,2),sep="")
pdf(paste(folder,"/absExprSummary_passing.pdf",sep=""), height=5, width=7)
verboseBoxplot(rAll,rNM,las=2,main="Between method corr",ylab="R",xlab="",notch=FALSE)
dev.off()

## Now plot all of these samples FROM BRAIN 1 (the test brain) individually
pdf(paste(folder,"/plotAllBrain1SSMIconversions_passing.pdf",sep=""),height=6,width=6)
plot(rArr[w1][order(rArr[w1])],pch=19,col="grey",ylim=c(0.6,1),ylab="Between-method correlation")
abline(h=0.95,lty="dashed",col="grey",lwd=2);  points(rLE[w1][order(rArr[w1])],pch=19);
dev.off()

## Show a before and after example (index 12, for consistency with paper)
pdf(paste(folder,"/beforeToAfterConversionPlotsBrain1_passing.pdf",sep=""),height=6,width=10.6)
i=12;   xy = c(-2,16);     par(mfrow=c(1,2))
verboseScatterplot(datNormRtmp[pass,i],datNormEtmp[pass,i],xlab="RSEM (TPM)",
 ylab="Array (intensity)",xlim=xy,ylim=xy,pch=19,cex=0.3,col="darkgrey");
abline(h=0);abline(v=0);abline(0,1,lty="dashed",col="black",lwd=2)
verboseScatterplot(datNormRtmp[pass,i],datTmpScale[pass,i],xlab="RSEM (TPM)",ylab="Array (mTPM)",
 xlim=xy,ylim=xy,pch=19,cex=0.3); abline(h=0);abline(v=0);abline(0,1,lty="dashed",col="grey",lwd=2)
dev.off()
}
###################### END OF LOOP ######################
```

\# This plot above, which is one of the outputs of the loop above for 16 samples, is used to make part of Additional File 7. Note that even with 16 samples we get much better data after scaling. This can actually be seen with as few as 5 samples, which is why I include 5 samples in the loop.

**## END OF CODE ##**
